# Causal role of serum metabolites in chronic periodontitis: A bidirectional Mendelian randomization and multi-omics integration study

**DOI:** 10.1097/MD.0000000000048615

**Published:** 2026-05-08

**Authors:** Xiaoli Li, Haohan Ye, Xiaofei Liu, Jun Tang, Rui Xiao

**Affiliations:** a Laboratory of Developmental Biology, Department of Genetics and Cell Biology, School of Basic Medical Sciences, Chongqing Medical University, Chongqing, China; b Department of Bioinformatics, School of Basic Medical Sciences, Chongqing Medical University, Chongqing, China; c Department of Rheumatology and Immunology, Hainan Hospital of Chinese PLA General Hospital, Sanya, Hainan, China; d Center for Experimental Teaching Management, Chongqing Medical University, Chongqing, China; e Department of Stomatology, The First Medical Center of PLA General Hospital, Beijing, China.

**Keywords:** causality, chronic periodontitis, Mendelian randomization analysis, metabolomics, multi-omics

## Abstract

Chronic periodontitis (CP) is a multifactorial inflammatory disease. Growing evidence links dysregulated serum metabolites to CP pathogenesis, yet their causal roles in disease progression remain poorly defined. We performed bidirectional Mendelian randomization (MR) analysis to evaluate causal relationships between 486 serum metabolites and CP. Inverse-variance weighted, MR-Egger, and weighted median methods were employed to mitigate research bias. Sensitivity was evaluated via the Cochran *Q* test, MR-Egger, and leave-one-out analysis. Metabolic pathway analysis (based on metabolomics data) was carried out via the MetaboAnalyst 6.0. We analyzed RNA-seq data to identify genes and mechanisms involved in serum metabolite regulation of CP development. Bidirectional MR identified 22 metabolites associated with CP risk, with 6 metabolites exhibiting protective effects and 16 linked to risk-increasing associations. Sensitivity analyses confirmed robustness across multiple MR methods, and reverse MR excluded reverse causality. Caffeine metabolism and lysine degradation emerged as the core pathways. Nineteen overlapping genes were identified via transcriptomic integration as key mediators. Enrichment analysis highlighted FoxO signaling and p53-mediated signaling. This study deepens the understanding of metabolic crosstalk and its role in CP pathogenesis, revealing novel biomarkers and therapeutic targets that inform diagnostic precision and therapeutic innovation. However, limitations such as sample size constraints should be noted. Future research could validate these findings in larger populations and explore the underlying biological mechanisms linking these metabolites to CP.

## 1. Introduction

Chronic periodontitis (CP), affecting 45% to 60% of adults globally, is a multifactorial inflammatory disease causing progressive periodontal tissue destruction and tooth loss.^[1,2]^ Emerging evidence underscores the bidirectional relationship between periodontal disease and systemic health, with periodontitis demonstrating significant correlations to conditions such as atherosclerotic cardiovascular disorders, chronic respiratory pathologies, metabolic dysregulation (e.g., diabetes mellitus), neurodegenerative diseases (e.g., Alzheimer disease), oncological processes, and obstetric complications, including preterm birth and preeclampsia.^[3–7]^ This systemic interplay highlights the necessity to elucidate the molecular mechanisms underlying periodontal pathogenesis, particularly the role of chronic inflammation, dysregulated immune responses, and microbial dysbiosis in driving extraoral manifestations. Such investigations are pivotal for developing diagnostic biomarkers and targeted therapeutic interventions, thereby addressing a critical gap in the prevention and management of both oral and systemic comorbidities.

While traditional risk factors such as smoking, diabetes, and genetic susceptibility have been well-documented, emerging evidence highlights the critical role of systemic metabolic dysregulation in modulating periodontal inflammation and tissue remodeling.^[8–10]^ Serum metabolites, as downstream mediators of genetic, environmental, and microbial interactions, may serve as key biomarkers reflecting pathophysiological states and therapeutic targets. Nuclear magnetic resonance (NMR)-based metabolomic profiling identified elevated short-chain fatty acids (e.g., hexanoic acid, butyric acid) in periodontitis saliva, correlating with diagnostic potential. These fatty acids promote IL-6/TNF-α release via TLR4/NF-κB while suppressing apoptosis through Bcl-2 upregulation, hindering tissue repair.^[11]^ Proton NMR revealed reduced acetone/methanol and elevated lactate in periodontitis.^[12]^ Metabolomic studies have identified dysregulated pathways in CP, including lipid peroxidation, amino acid catabolism, and energy metabolism.^[13–15]^ However, causal relationships between specific metabolites and CP remain poorly elucidated due to confounding biases inherent in observational designs.

Mendelian randomization (MR), a genetic epidemiological method using genome-wide association study (GWAS) summary statistics, avoids reverse causality and confounding in traditional observational studies.^[16,17]^ Which prior MR studies have explored serum metabolite-CP associations,^[18,19]^ large-scale, systematic evaluations of serum metabolites-CP causality remain unexplored, leaving critical gaps in understanding metabolic dysregulation’s role in periodontal pathogenesis. Here, we hypothesize that specific serum metabolites (e.g., lipid derivatives and amino acids) exhibit causal relationships with CP risk, with distinct protective or risk-enhancing effects. To test this, we perform bidirectional MR analysis integrating multi-omics data to quantify metabolite-CP causal effects and identify key pathways through pathway enrichment. Notably, this is the first large-scale MR study addressing serum metabolite-CP causal relationship, thereby overcoming limitations of prior observational and small-scale metabolomic research. This investigation advances metabolomic characterization of CP and provides mechanistic insights for developing metabolite-targeted therapeutic strategies.

## 2. Materials and methods

### 2.1. Study design

**Figure 1.**
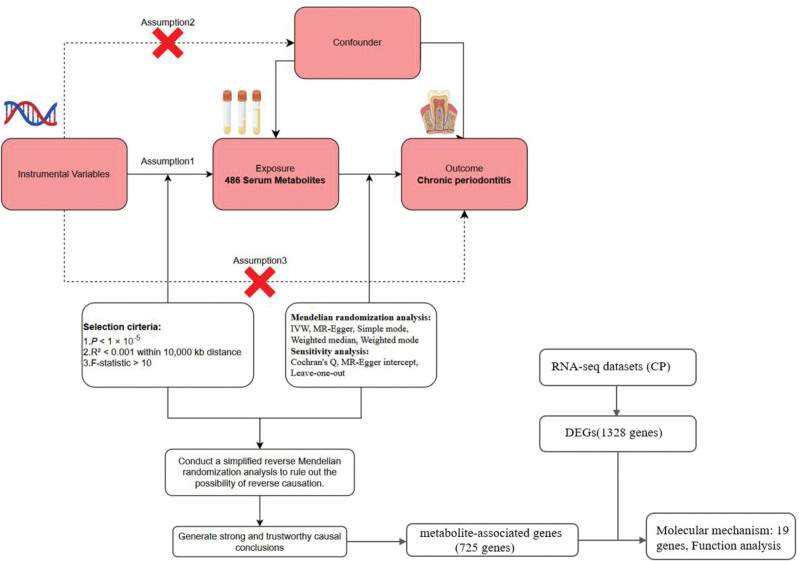
Schematic overview of the study design. CP = chronic periodontitis, DEG = differentially expressed gene, IVW = inverse-variance weighted.

This study integrated MR and multi-omics analysis to identify causal serum metabolites and mechanisms underlying CP. As shown in Figure 1. A 2-sample MR framework was employed using GWAS summary statistics from multiple cohorts to evaluate causal relationships between 486 metabolites and CP. Instrumental variables (IVs) were selected based on 3 criteria: strong genetic association with metabolites (*F*-statistics > 10), no overlap with CP-associated loci, and exclusivity of effect through metabolites. Bidirectional MR was implemented using the TwoSampleMR R package (v0.5.11), with inverse-variance weighted (IVW), MR-Egger, and weighted median (WM) methods to estimate causal effects. Sensitivity analyses included the Cochran *Q* statistic, MR-Egger, and leave-one-out (LOO) validation to assess instrument validity. We systematically assessed metabolic pathway enrichment via MetaboAnalyst 6.0 to identify dysregulated pathways associated with disease phenotypes. Multi-omics integration merged transcriptomic profiles from CP patients with metabolomic datasets to identify overlapping genes, and functionally annotated these central genes. Ethical review was waived for this secondary analysis as all datasets were sourced from publicly available repositories with established ethical approvals, ensuring compliance with the Declaration of Helsinki and participant consent requirements. The analysis was conducted using R 4.3.3.

### 2.2. Data source

Serum metabolomic data were sourced from Shin et al’s metabolomic GWAS, analyzing 7824 European participants (1768 German; 6056 UK) via MS-based metabolomics, identifying 486 metabolites (309 chemically defined) linked to 2.1M GWAS loci.^[20]^ For CP, we used FinnGen R11 data (K11_PERIODON_CHRON dataset: 5364 cases vs 288,472 controls) with GWAS summary statistics.^[21]^

### 2.3. Selection of IVs

IVs were systematically identified through a 3-stage pipeline: Genome-wide association analysis (*P* < 1 × 10^−5^) identified metabolite-associated single-nucleotide polymorphisms (SNPs). Linkage disequilibrium pruning (*r*^2^ < 0.001, 10,00 kb window) eliminated correlated variants.^[22]^ *F*-statistic validation (*F* > 10) ensured instrument strength,^[23]^ calculated as *F* = *R*^2^ × (n − *k* − 1)/(*k* × [1 − *R*^2^]), where *R*^2^ = 2 × (1 − MAF) × MAF × β^2^ (n: sample size, *k*: IV count, MAF: minor allele frequency, β: effect size).^[24]^

### 2.4. MR and sensitivity analysis

MR evaluated causal relationships between serum metabolites and CP using 3 analytical frameworks: IVW regression (assuming linearity/monotonicity) for primary estimates, MR-Egger for pleiotropy adjustment, and WM for robustness.^[25,26]^ To mitigate horizontal pleiotropy and weak instrument bias, we implemented MR-Egger regression and WM estimation. Bidirectional MR (metabolites → CP and CP → metabolites) confirmed causal directionality. Consistency across IVW/MR-Egger/WM estimates (overlap in 95% confidence interval [CI]) and absence of pleiotropic outliers (MR-Egger intercept, *P* > .05) validated causal specificity. This multi-method approach balanced statistical rigor with mechanistic interpretability. Heterogeneity was assessed via the Cochran *Q* test (*P* < .05 indicates significant variant-specific effects).^[27]^ MR-Egger regression detected directional pleiotropy (nonzero intercept, *P* < .05),^[28,29]^ while LOO analysis evaluated outlier sensitivity by iteratively excluding individual SNPs.^[28]^ This multi-tiered approach balanced methodological rigor with translational relevance, mitigating risks of confounding and instrument validity breaches.

### 2.5. Metabolic pathway analyses

Metabolic pathway analysis was performed using MetaboAnalyst 6.0 (https://www.metaboanalyst.ca/) with Kyoto Encyclopedia of Genes and Genomes (KEGG) database, applying hypergeometric tests (FDR < 0.05) to identify significantly enriched pathways (*P* < .05) associated with CP progression.

### 2.6. Mapping SNPs to genes and identification of differentially expressed genes

To identify cis-regulatory mechanisms linking serum metabolites to CP risk, we mapped genetic variants to target genes using SNPnexus (https://www.snp-nexus.org), incorporating functional annotations for promoters, enhancers, and untranslated regions.^[30]^ Transcriptomic data were derived from GSE171213 (Illumina RNA-seq), comprising 4 CP cases and 5 healthy controls. Differentially expressed genes (DEGs) were identified via limma R package (v3.58.1) using thresholds of Log_2_ fold change >1 and *P* < .05.^[31]^

### 2.7. Identification and functional enrichment analysis of overlapping genes

To identify shared genetic mediators between serum metabolites and CP risk, we performed a systematic convergence analysis of metabolite-associated genes and DEGs. This involved annotating metabolite-related genes using pathway enrichment tools, intersecting these genes with DEGs from gingival tissue transcriptomics, and prioritizing overlapping genes based on statistical significance and biological relevance. Functional enrichment analysis was conducted using the “ClusterProfiler” R package (version 4.10.1) to elucidate functional interaction networks and pathway mechanisms.^[32]^

## 3. Results

### 3.1. Strength of the IVs

To elucidate the causal relationship between serum metabolites and CP pathogenesis, we implemented a dual-sample MR framework leveraging GWAS summary statistics. Among 486 metabolites evaluated, each demonstrated association with 3 to 307 SNPs functioning as IVs. Notably, fructose and ergothioneine exhibited minimal IV representation (3 SNPs each), whereas 2-methoxyacetaminophen sulfate displayed the highest IV density (307 SNPs). Rigorous IV quality control was performed by enforcing an *F*-statistic threshold >10 for all IVs (minimum observed *F* = 17.45), surpassing the conventional robustness criterion in MR methodologies.

### 3.2. MR analysis results

**Figure 2.**
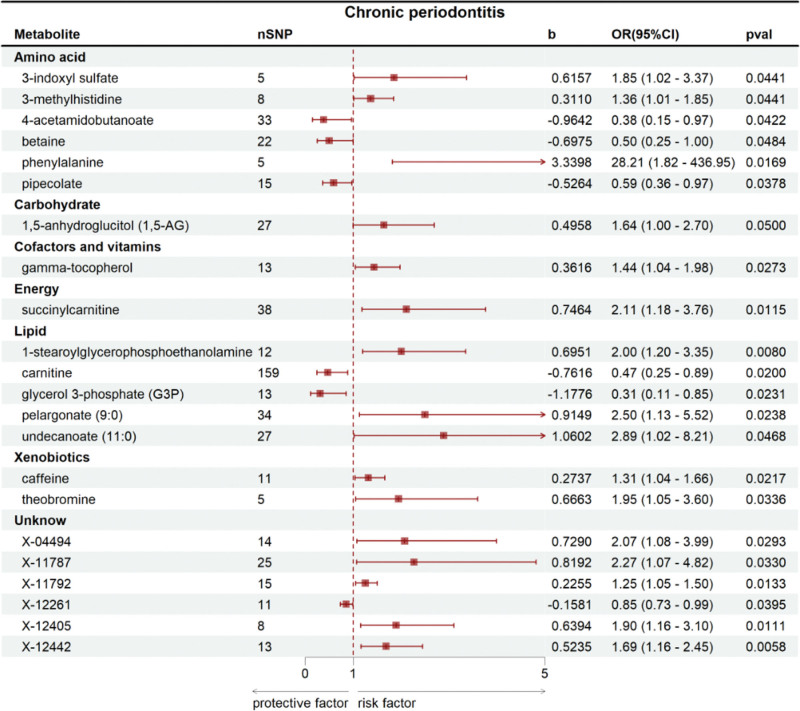
MR forest plots depicting causal associations of serum metabolites with CP risk. CI = confidence interval, CP = chronic periodontitis, MR = Mendelian randomization, OR = odds ratio, SNP = single-nucleotide polymorphisms.

**Figure 3.**
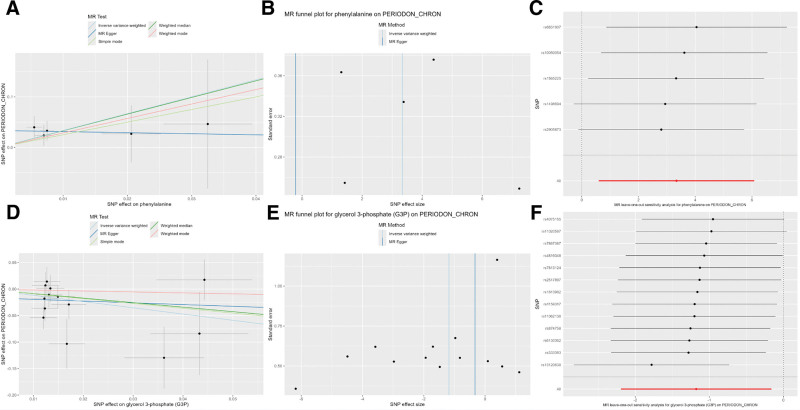
Genetic instrument-outcome associations for top-ranked risk and protective serum metabolites with CP, presented as scatter plots (A, D), funnel plots (B, E), and LOO sensitivity plots (C, F). (A) Scatter plot for top-risk metabolite. (B) Funnel plot for top-risk metabolite. (C) LOO plot for top-risk metabolite. (D) Scatter plot for top-protective metabolite. (E) Funnel plot for top-protective metabolite. (F) LOO plot for top-protective metabolite. CP = chronic periodontitis, LOO = leave-one-out, MR = Mendelian randomization, SNP = single-nucleotide polymorphisms.

Using bidirectional MR with 486 serum metabolites, we identified 22 metabolites significantly associated with CP risk in IVW analysis (*P* < .05). These comprised 16 identified metabolites (with known biological functions) and 6 novel/unannotated metabolites (lacking functional annotations). Key findings are summarized in Figure 2 and Table 1. Among these, 6 metabolites exhibited protective effects (5 identified and 1 unidentified): glycerol 3-phosphate (G3P) showed the strongest inverse association (odds ratio [OR] = 0.3080, 95% CI = 0.1115–0.8508, *P* = .0231), followed by 4-acetamidobutanoate (OR = 0.3813, 95% CI = 0.1504–0.9667, *P* = .0422), carnitine (OR = 0.4669, 95% CI = 0.2458–0.8870, *P* = .0200), betaine (OR = 0.4979, 95% CI = 0.2491–0.9951, *P* = .0484), pipecolate (OR = 0.5907, 95% CI = 0.3595–0.9708, *P* = .0378), and X-12261 (OR = 0.8538, 95% CI = 0.7345–0.9925, *P* = .0395). Conversely, 16 metabolites were positively associated with CP risk (11 identified and 5 unidentified), including caffeine (OR = 1.3148, 95% CI = 1.0407–1.6609, *P* = .0217), 3-methylhistidine (OR = 1.3648, 95% CI = 1.0083–1.8473, *P* = .0441), gamma-tocopherol (OR = 1.4356, 95% CI = 1.0414–1.9791, *P* = .0273), 1,5-anhydroglucitol (1,5-AG; OR = 1.6418, 95% CI = 1.0000–2.6953, *P* = .0500), 3-indoxyl sulfate (OR = 1.8510, 95% CI = 1.0165–3.3709, *P* = .0441), theobromine (OR = 1.9471, 95% CI = 1.0532–3.5997, *P* = .0336), 1-stearoylglycerophosphoethanolamine (OR = 2.0040, 95% CI = 1.1986–3.3505, *P* = .0080), succinylcarnitine (OR = 2.1095, 95% CI = 1.1825–3.7631, *P* = .0115), pelargonate (9:0; OR = 2.4965, 95% CI = 1.1295–5.5181, *P* = .0238), undecanoate (11:0; OR = 2.8870, 95% CI = 1.0153–8.2091, *P* = .0468), and phenylalanine (OR = 28.2144, 95% CI = 1.8218–436.9547, *P* = .0169), X-11792 (OR = 1.2529, 95% CI = 1.0480–1.4979, *P* = .0133), X-12442 (OR = 1.6879, 95% CI = 1.1638–2.4480, *P* = .0058), X-12405 (OR = 1.8953, 95% CI = 1.1574–3.1038, *P* = .0111), X-04494 (OR = 2.0731, 95% CI = 1.0763–3.9932, *P* = .0293), X-11787 (OR = 2.2688, 95% CI = 1.0684–4.8177, *P* = .0330). Notably, phenylalanine had the highest risk magnitude (Fig. 3 and Fig. S1, Supplemental Digital Content).

**Table 1:** MR analysis of causal effects of 22 metabolites on CP risk with heterogeneity and horizontal pleiotropy assessment.

Metabolite	nSNP	Cochran *Q* test		MR-Egger intercept	
		IVW	MR-Egger	Egger intercept	*P*
1,5-anhydroglucitol (1,5-AG)	27	0.5812	0.5387	−0.0056	.6282
1-stearoylglycerophosphoethanolamine	12	0.6743	0.6470	−0.0137	.4503
3-indoxyl sulfate	5	0.4921	0.3899	−0.0603	.5745
3-methylhistidine	8	0.5569	0.4406	0.0023	.9337
4-acetamidobutanoate	33	0.0100	0.0088	−0.0083	.5141
Betaine	22	0.2401	0.1972	0.0027	.8405
Caffeine	11	0.2557	0.2986	0.0386	.2516
Carnitine	159	0.9412	0.9458	−0.0064	.2170
Gamma-tocopherol	13	0.5780	0.5685	0.0129	.3755
Glycerol 3-phosphate (G3P)	13	0.2015	0.1848	−0.0161	.4507
Pelargonate (9:0)	34	0.2287	0.1968	0.0045	.7704
Phenylalanine	5	0.7583	0.9602	0.0334	.2979
Pipecolate	15	0.8667	0.8835	−0.0196	.3201
Succinylcarnitine	38	0.6155	0.6174	−0.0077	.3226
Theobromine	5	0.8913	0.7745	0.0074	.9326
Undecanoate (11:0)	27	0.1596	0.2012	0.0188	.1700
X-04494	14	0.8672	0.8174	−0.0025	.8275
X-11787	25	0.9291	0.9265	−0.0085	.4141
X-11792	15	0.9493	0.9380	−0.0118	.5487
X-12261	11	0.5602	0.4658	0.0015	.9219
X-12405	8	0.5565	0.7831	−0.0443	.1543
X-12442	13	0.9912	0.9835	−0.0024	.8703

CP = chronic periodontitis, IVW = inverse-variance weighted, MR = Mendelian randomization, SNP = single-nucleotide polymorphisms.

### 3.3. Sensitivity and reverse causality analysis results

Sensitivity analyses confirmed MR robustness. The MR-Egger intercept demonstrated no evidence of pleiotropic bias (*P* > .05; Fig. S2, Supplemental Digital Content). The Cochran *Q* test indicated no heterogeneity (Table 1). LOO analysis demonstrated causal stability (Fig. S3, Supplemental Digital Content). Reverse MR analysis excluded reverse causation (all *P* > .05; Table S1, Supplemental Digital Content), thereby validating metabolite → CP causality.

### 3.4. Results of metabolic pathway analyses

**Figure 4.**
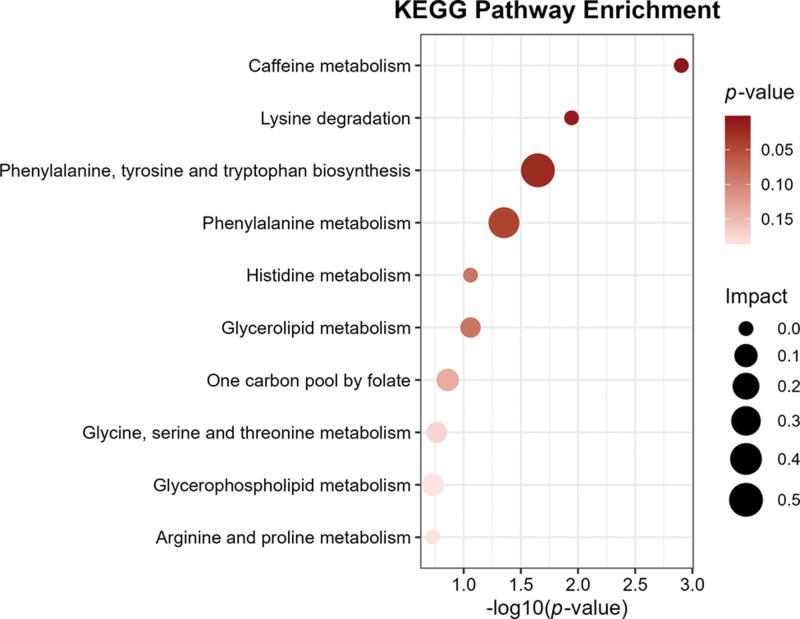
Displays the enrichment pathways of metabolites in KEGG. KEGG = Kyoto Encyclopedia of Genes and Genomes.

Metabolic pathway analysis revealed that caffeine metabolism and lysine degradation were significantly enriched in CP, with FDR-adjusted *P* < .05, establishing these pathways as pivotal contributors to CP-associated metabolic reprogramming, as shown in Figure 4 and Table S2, Supplemental Digital Content.

### 3.5. Mapping SNPs to genes and DEGs identification of CP

**Figure 5.**
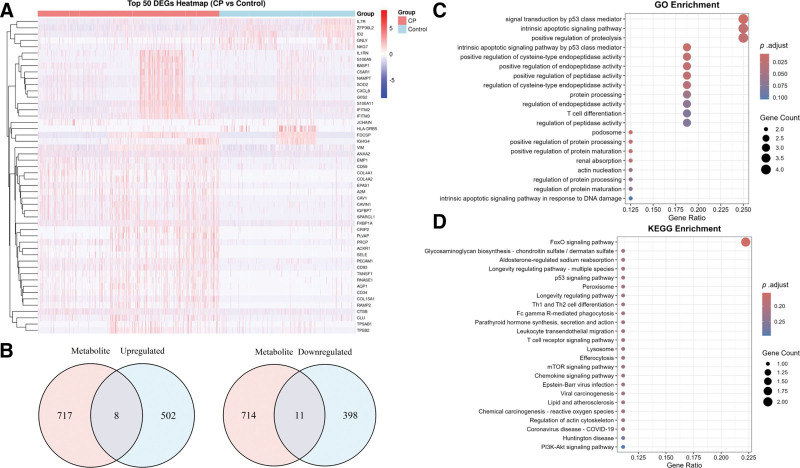
Identification and functional analysis of overlapping genes between serum metabolite-associated genes and DEGs in CP. (A) A heatmap of the top 50 DEGs in CP. (B) Venn diagram of 24 overlapping genes between DEGs and metabolite-associated genes. (C) GO enrichment for overlapping genes. (D) KEGG pathway enrichment for overlapping genes. CP = chronic periodontitis, DEG = differentially expressed gene, GO = Gene Ontology, KEGG = Kyoto Encyclopedia of Genes and Genomes.

Utilizing the SNPnexus web-based platform, we curated a compendium of 725 metabolite-associated genes. Through differential expression analysis of RNA-seq data from 4 healthy individuals and 5 CP patients, we identified 919 significantly dysregulated genes (510 upregulated and 409 downregulated, FDR < 0.05). Hierarchical clustering of these transcripts unveiled distinct molecular signatures, depicted via a heatmap with *z*-score normalized expression values (Fig. 5A).

### 3.6. Identification and pathway enrichment analysis of metabolite-associated overlapping genes in CP

To identify genes common to both metabolite-associated genes and DEGs, we performed a set intersection analysis and visualized overlaps via Venn diagrams. This revealed 8 overlapping genes between upregulated DEGs and metabolite-associated genes, and 11 overlapping genes between downregulated DEGs and metabolite-associated genes (Fig. 5B). To decode the functional significance of these overlapping genes in CP pathogenesis, we conducted Gene Ontology (GO) and KEGG pathway enrichment analyses, focusing on terms with FDR < 0.05 and enrichment fold change >1.5. GO results revealed strong enrichment in regulation of signal transduction by p53 class mediator, intrinsic apoptotic signaling pathway, and positive regulation of proteolysis (Fig. 5C). KEGG analysis further highlighted their involvement in FoxO signaling pathway, glycosaminoglycan biosynthesis-chondroitin sulfate/dermatan sulfate, aldosterone-regulated sodium reabsorption (Fig. 5D).

## 4. Discussion

This MR study systematically evaluated the causal relationship between 486 serum metabolites and the risk of CP. Among the 22 metabolites (16 known and 6 unknown) with significant causal relationships (*P* < .05), 6 metabolites showed protective effects, while 16 metabolites were associated with increased CP risk. Metabolic pathway analysis identified caffeine metabolism and lysine degradation as central dysregulated pathways in CP. Integrated transcriptomic analysis identified 19 overlapping genes bridging metabolite-associated pathways and CP-related transcriptional dysregulation, significantly enriched in pathways linking metabolic stress to immune-apoptotic crosstalk. These genes act as central regulators of metabolite-CP risk interplay, positioning them as critical nodes for understanding how biochemical perturbations drive inflammatory tissue destruction in CP. These findings underscore the role of metabolite-driven transcriptional rewiring in CP pathogenesis, highlighting potential diagnostic biomarkers and therapeutic avenues targeting metabolic-immune checkpoints.

Our study identified phenylalanine as the strongest risk factor for CP, where it promotes gingival inflammation and osteoclast activation via kynurenine pathway-driven pro-inflammatory cytokine production (e.g., IL-6, TNF-α). NMR metabolomic analysis revealed distinct salivary profiles in CP/aggressive periodontitis patients: pyruvate, lactate, and N-acetyl metabolites were significantly reduced, while phenylalanine, proline, and tyrosine were elevated.^[33]^ Baima et al identified differential metabolites in saliva samples from periodontitis patients. Metabolic pathway enrichment analysis revealed that phenylalanine, tyrosine, and tryptophan biosynthesis pathways exhibited the most significant alterations compared to healthy controls. The increase in concentration of amino acids as protein breakdown products may be related to the damage of inflammation to periodontal tissue.^[34]^ Our study further identified phenylalanine as the strongest risk factor for CP. Consistent with previous findings, this suggests that phenylalanine may drive periodontal inflammation by promoting bacterial proliferation and modulating host immune responses (e.g., releasing pro-inflammatory cytokines). Further experimental validations are warranted to confirm these mechanisms.

Our MR analysis identifies G3P as a robust protective metabolite against CP. As a pivotal metabolic hub, G3P orchestrates flux through glycolytic and gluconeogenic pathways, which underpin cellular energetic equilibrium. In meningiomas, aberrant rewiring of glucose metabolism manifests as hyperactivated glycolysis, fueling the unchecked proliferation characteristic of these tumors.^[35]^ While G3P is a canonical intermediate in glycolysis and phospholipid synthesis, emerging evidence suggests its broader roles. In the context of chronic kidney disease, G3P exerts pleiotropic effects on metabolic and mineral homeostasis. Specifically, G3P augments hepatic gluconeogenesis via allosteric activation of fructose-1,6-bisphosphatase, while simultaneously alleviating oxidative stress through upregulation of glutathione peroxidase activity in renal tubular cells. This dual action preserves cellular bioenergetic integrity and delays fibrosis progression.^[36,37]^ Furthermore, G3P orchestrates FGF23 transcription in osteocytes, disrupting renal phosphate reabsorption and promoting vascular calcification.^[38,39]^ Notably, while prior investigations have predominantly focused on the roles of G3P in cardiovascular diseases, renal pathologies, and diabetes mellitus, direct evidence elucidating its involvement in periodontitis remains scarce. Emerging evidence, however, suggests that G3P may mitigate periodontal disease progression through multifaceted mechanisms.^[40]^ This positions G3P as a promising biomarker candidate and therapeutic target for periodontitis intervention, warranting rigorous exploration in future studies.

Metabolomic profiling revealed most significant dysregulation of caffeine metabolism and lysine degradation pathways in CP. Caffeine, a methylxanthine alkaloid synthesized in *Coffea arabica* and *Camellia sinensis*.^[41]^ Caffeine has been implicated in osteoarthritis through dose-dependent effects on joint cartilage during critical developmental periods. MR analysis further confirms this causal relationship, with genetically predicted caffeine exposure increasing knee osteoarthritis risk.^[42,43]^ Our findings reveal that caffeine metabolism constitutes a significantly enriched pathway in CP, suggesting its pivotal role in oral homeostasis. Future research should prioritize pharmacogenomic-guided caffeine modulation to establish personalized intake thresholds for periodontal disease management. The other metabolic pathway that mediates PC is the lysine degradation pathway. Emerging evidence positions the lysine degradation pathway as a critical modulator in the pathogenesis of prediabetic metabolic syndrome and type 2 diabetes.^[44]^ Changes in the concentrations of metabolites for lysine degradation correlated with prediabetic state in an animal study.^[45]^ Cumulative evidence indicates that the lysine degradation pathway exhibits a robust correlation with aberrant glucose homeostasis, positioning it as a modifiable nutritional intervention target for mitigating CP progression.

Integrative metabolomic-transcriptomic analysis identified 19 overlapping genes bridging metabolite-associated pathways and CP-related transcriptional dysregulation. Among the identified DEGs, *SOD2* and *ADAM8* have emerged as pivotal regulators implicated in CP pathogenesis.^[46,47]^ Their dysregulated expression highlights a potential link to disease progression. Stratifying *SOD2* and *ADAM8* expression levels in CP patients may therefore improve risk stratification for adverse clinical outcomes in CP. Notably, pathway enrichment analysis identified critical molecular mechanisms bridging metabolite dysregulation and CP. GO analysis revealed that these genes are functionally linked to p53-mediated stress responses, intrinsic apoptosis, and proteolysis, suggesting that metabolic dysregulation may disrupt cellular stress adaptation, trigger programmed cell death, and promote extracellular matrix (ECM) degradation. Pathway analysis further implied FoxO signaling and glycosaminoglycan biosynthesis pathways modulate inflammatory responses and ECM remodeling. Collectively, these findings unveil a metabolic-immune-apoptotic axis where serum metabolites (e.g., phenylalanine, glycerol 3-phosphate) activate FoxO and p53 pathways, triggering a cascade of events including GAG degradation, proteolytic ECM remodeling, and p53-mediated apoptosis. Such dysregulation establishes a self-perpetuating cycle of inflammation and tissue destruction, offering a mechanistic framework for understanding how metabolite imbalances propagate CP progression. While these findings align with prior studies linking metabolic stress to periodontal inflammation,^[48,49]^ the current data do not establish direct causality between specific metabolites (e.g., phenylalanine) and pathway activation. Further experimental validation is required to confirm causality and elucidate the specific metabolite-pathway interactions.

This study has several limitations worthy of note. First, the reliability of MR findings hinges on the strength of genetic instruments in explaining exposure variability, while we used a large biobank dataset, but metabolites with small-effect sizes may still be underpowered, limiting the robustness of associations. Second, while bidirectional MR supports a causal role for serum metabolites in CP, residual confounding cannot be fully ruled out without cross-validation using orthogonal methods (e.g., in vitro assays of metabolite effects on periodontal cells or in vivo animal models of CP). Third, the transcriptomic analysis was based on a relatively small sample size, which may limit the statistical power to detect subtle gene-metabolite interactions. In addition, the lack of replication in independent cohorts means that the mechanistic findings remain exploratory and require further validation. Fourth, functional evidence for the identified mediators (e.g., *SOD2*, *ADAM8*) and enriched pathways (FoxO signaling, p53-mediated apoptosis) remains limited-current results are based solely on genetic associations, not experimental validation. To address these limitations, future research should focus on 4 key areas. First, larger, multi-ancestry cohorts (including underrepresented populations such as Asian or African descent) would enhance the generalizability of genetic instrument effects and reduce bias from population stratification. Second, longitudinal studies with extended follow-up are needed to assess the long-term effects of serum metabolites on CP progression and treatment response, while multicenter validation using standardized metabolomic platforms would ensure the reproducibility of key metabolite associations across different populations. Third, expand the transcriptomic dataset through collaborative consortia (e.g., the Periodontitis Omics Consortium) and conduct replication analyses in independent populations (e.g., East Asian and European cohorts) to assess generalizability. Fourth, integrating multi-omics data (proteomics, epigenomics) with MR results would provide a more comprehensive view of how metabolites interact with genetic and environmental factors to drive CP pathogenesis. In addition, experimental studies (e.g., CRISPR-mediated knockdown of *SOD2/ADAM8* in CP-relevant cell lines) are critical to confirm the functional roles of identified mediators and pathways.

Our findings have important clinical implications. First, the identified causal metabolites could serve as novel diagnostic biomarkers for CP, enabling earlier detection and risk stratification. Second, the enriched pathways (FoxO signaling, p53-mediated apoptosis) provide potential therapeutic targets for CP management. Targeting these pathways (e.g., using FoxO inhibitors or p53 activators) could mitigate inflammatory tissue destruction and slow CP progression. Finally, our study highlights the importance of considering metabolic dysregulation in CP pathogenesis, suggesting that metabolite-based interventions (e.g., dietary modifications, metabolic modulators) could complement existing treatments to improve clinical outcomes.

## 5. Conclusion

This MR study identified 22 serum metabolites with causal associations to CP. The robust causal evidence highlights the potential of targeting metabolic pathways for CP prevention or therapy. Pathway analysis further pinpointed caffeine metabolism and lysine degradation as core metabolic dysregulations driving these gene-metabolite interactions. Transcriptomic integration revealed 19 overlapping genes as key mediators, linking metabolic dysregulation to FoxO signaling, glycosaminoglycan metabolism, and p53-mediated apoptosis. These findings elucidate a possible multi-omics framework where metabolites drive CP pathogenesis through gene-regulated immune and apoptotic mechanisms, offering potential integrated targets for precision prevention and therapy. However, limitations include potential residual confounding in bidirectional MR, limited statistical power for small-effect metabolites, small transcriptomic sample size, and lack of functional validation. Future studies should expand multi-ancestry cohorts, conduct longitudinal and multicenter validations, and integrate multi-omics with experimental approaches to confirm mechanisms.

## Acknowledgments

We are indebted to the researchers who pioneered the initial GWAS research and openly shared their summary statistics.

## Author contributions

**Conceptualization:** Xiaoli Li.

**Formal analysis:** Xiaoli Li, Haohan Ye.

**Funding acquisition:** Rui Xiao.

**Methodology:** Xiaoli Li.

**Project administration:** Xiaoli Li.

**Resources:** Xiaoli Li.

**Supervision:** Xiaoli Li, Rui Xiao.

**Validation:** Xiaoli Li, Haohan Ye.

**Writing – original draft:** Xiaoli Li.

**Writing – review & editing:** Xiaoli Li, Rui Xiao.

**Data curation:** Haohan Ye.

**Investigation:** Haohan Ye, Xiaofei Liu, Jun Tang.

**Software:** Haohan Ye.

**Visualization:** Haohan Ye.










