# Supplementary material for: Causal role of serum metabolites in chronic periodontitis: A bidirectional Mendelian randomization and multi-omics integration study
Source: Medicine (Baltimore). 2026 May 8;105(19):e48615. doi: 10.1097/MD.0000000000048615 (PMC13166884; doi:10.1097/MD.0000000000048615)

## Slide 1
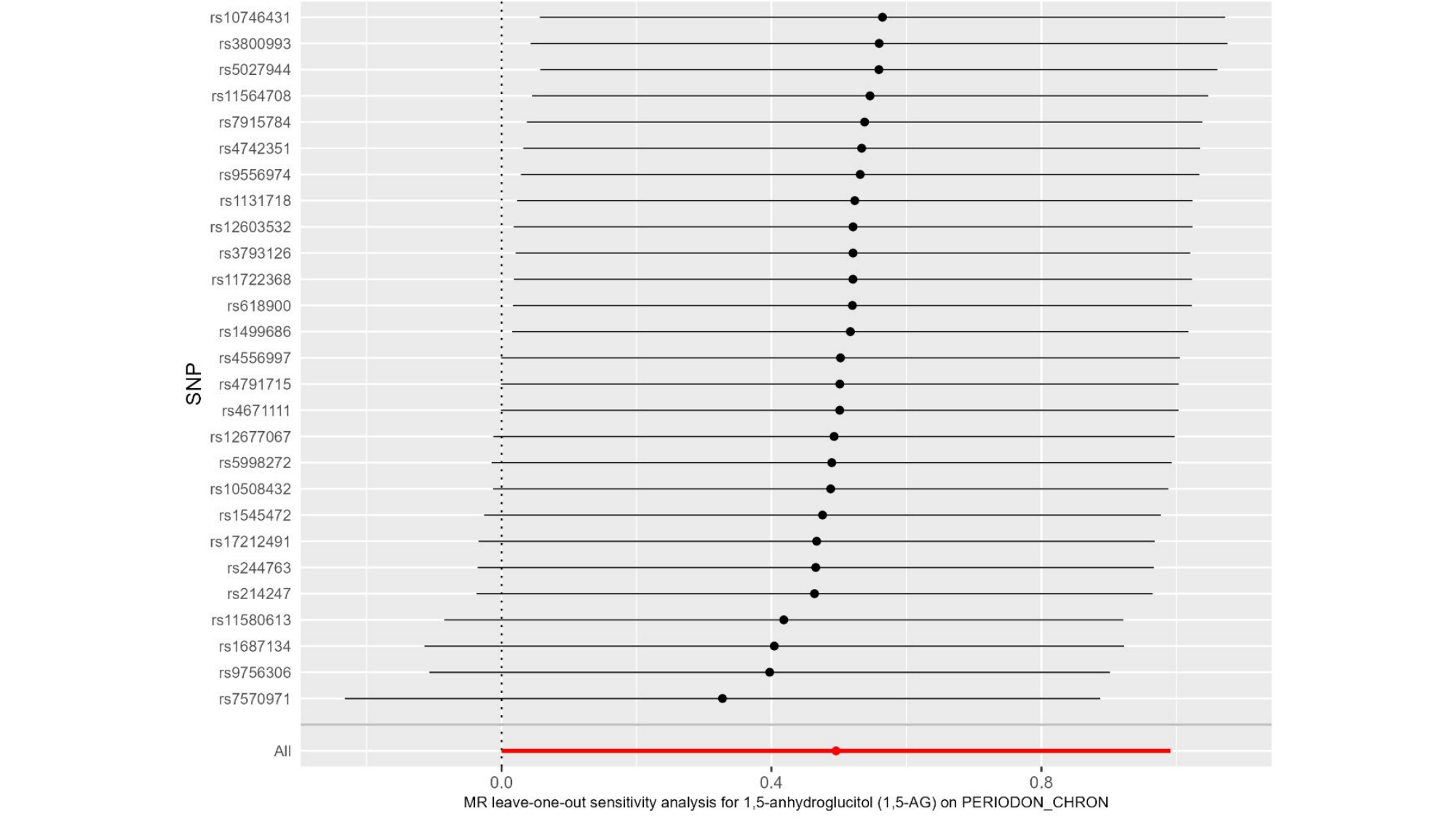

## Slide 2
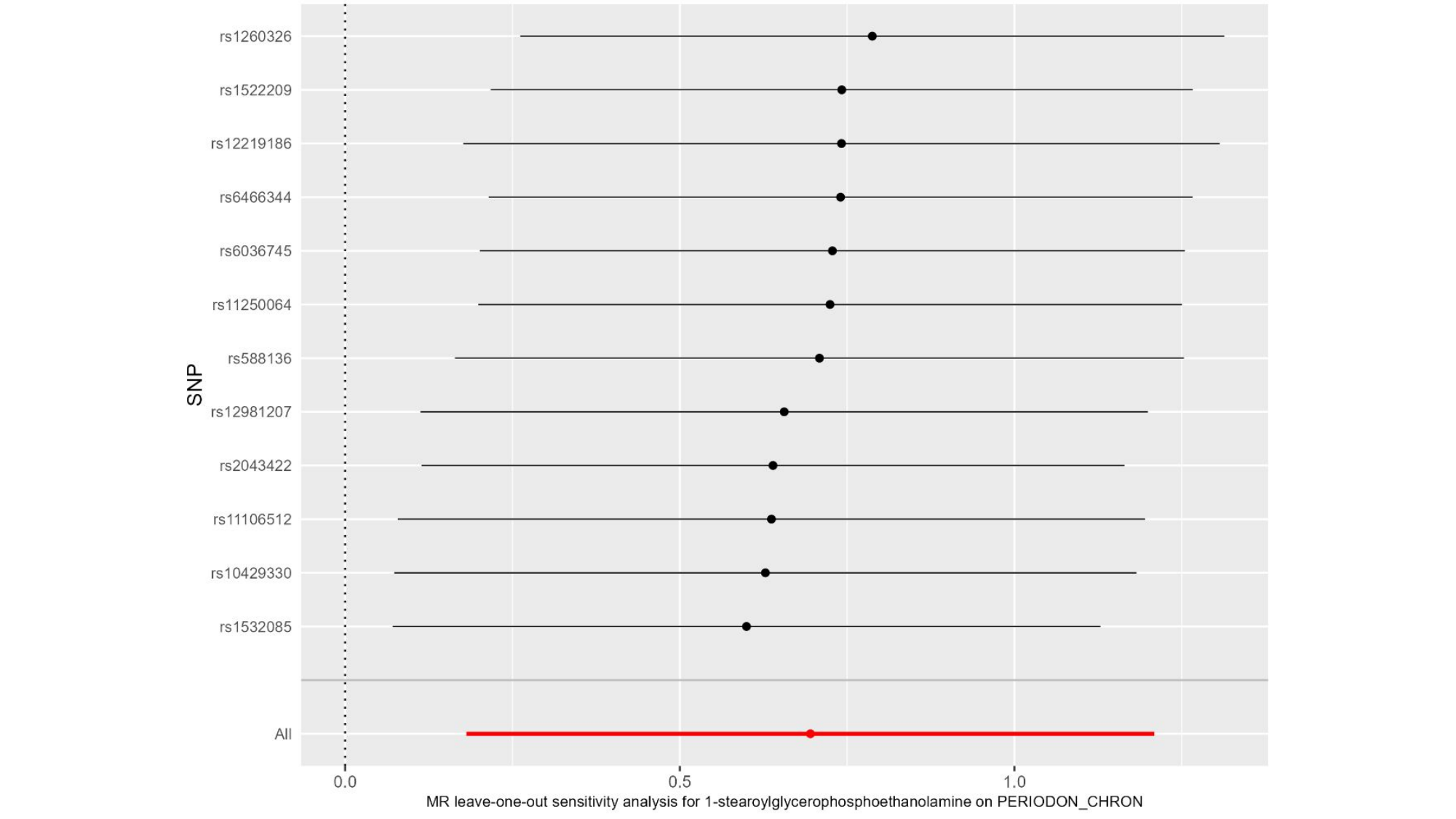

## Slide 3
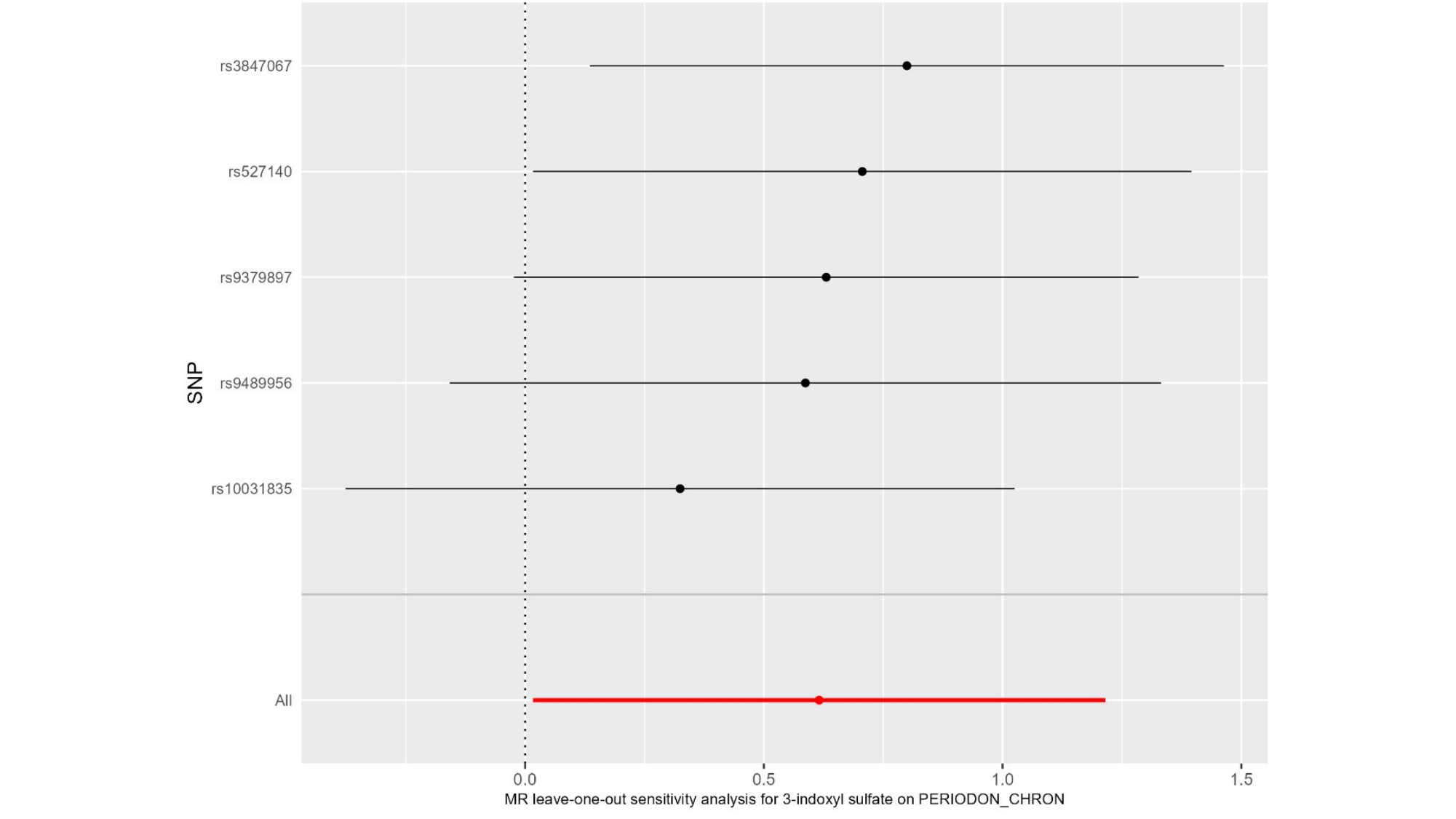

## Slide 4
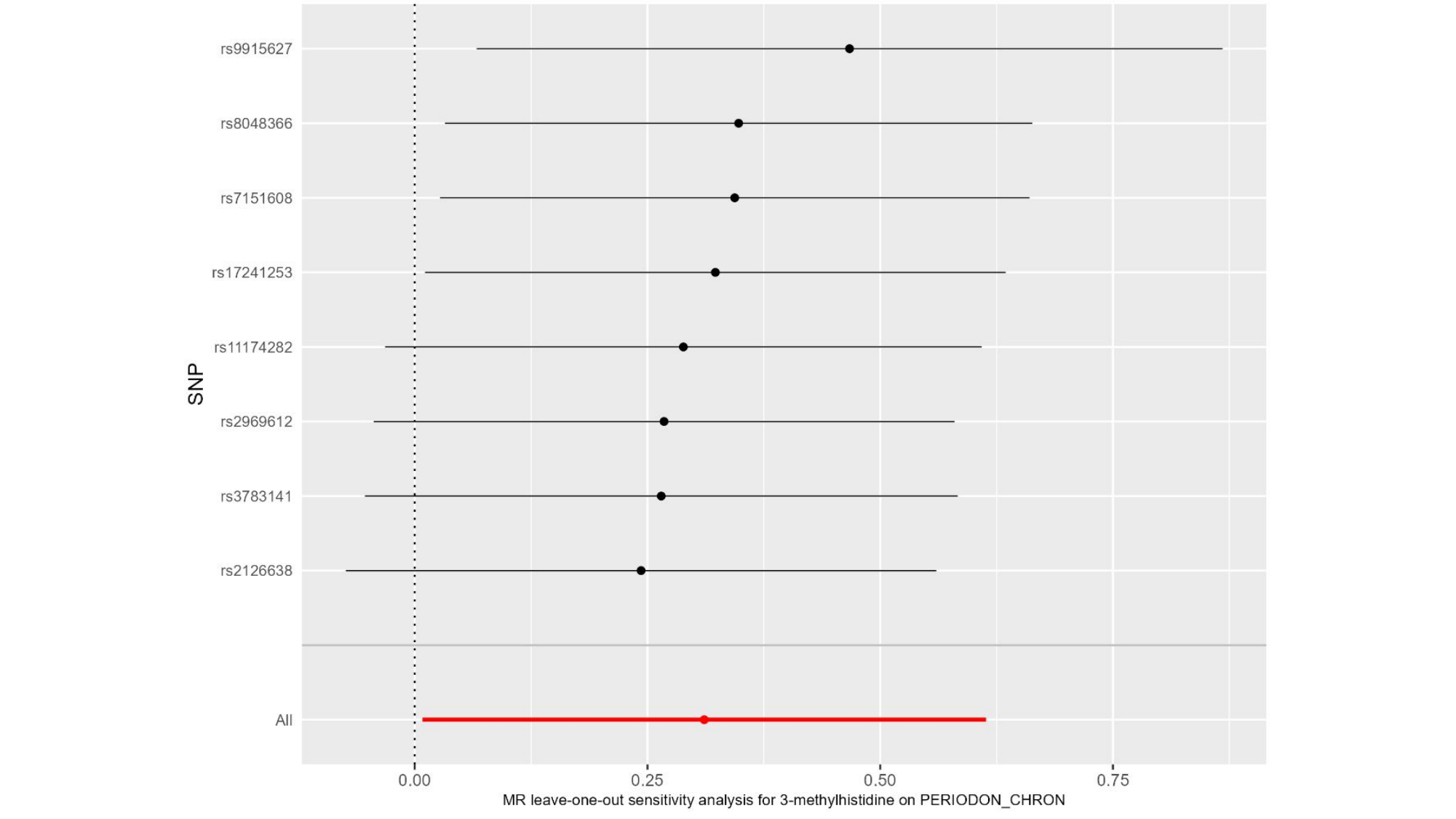

## Slide 5
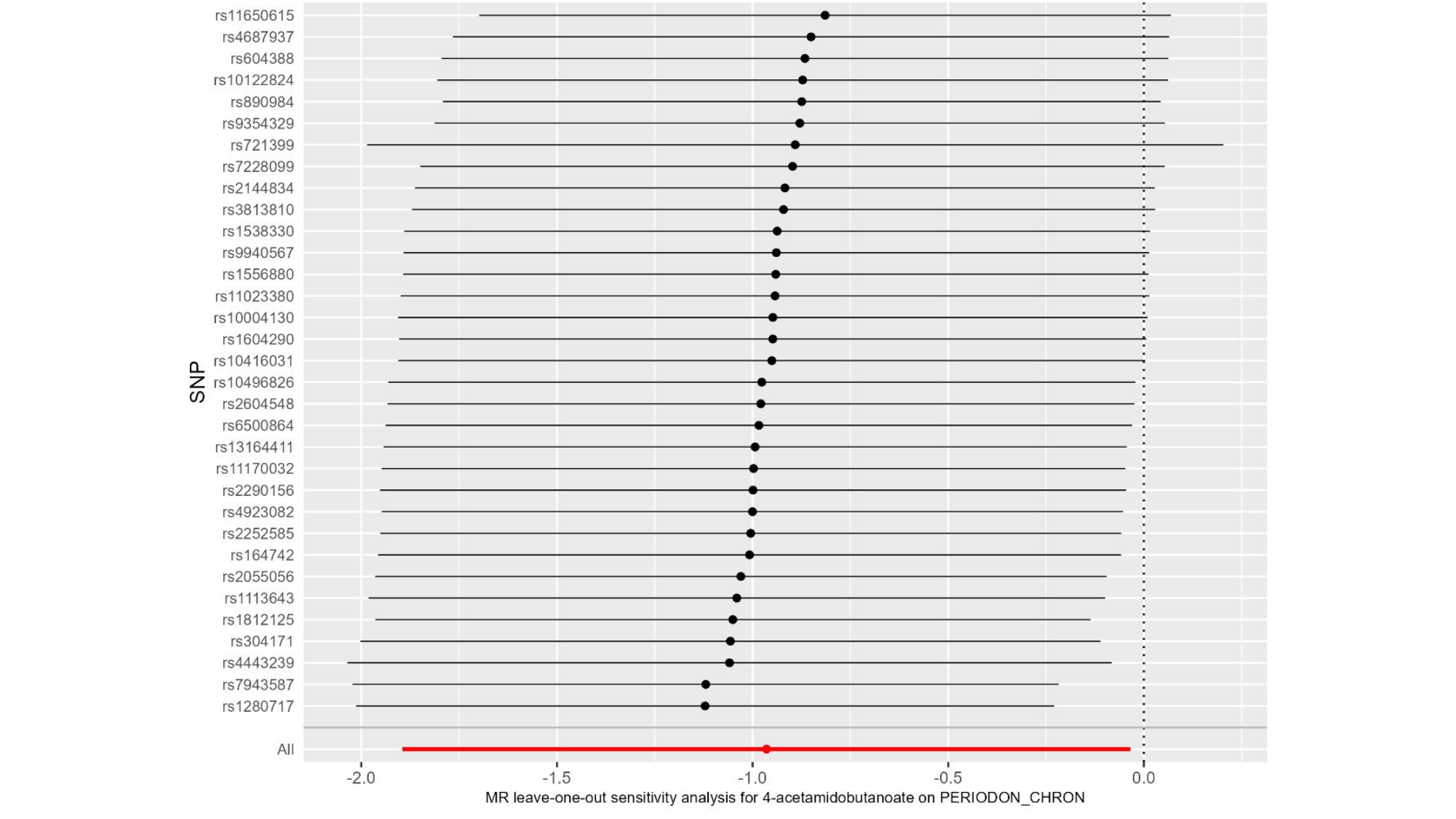

## Slide 6
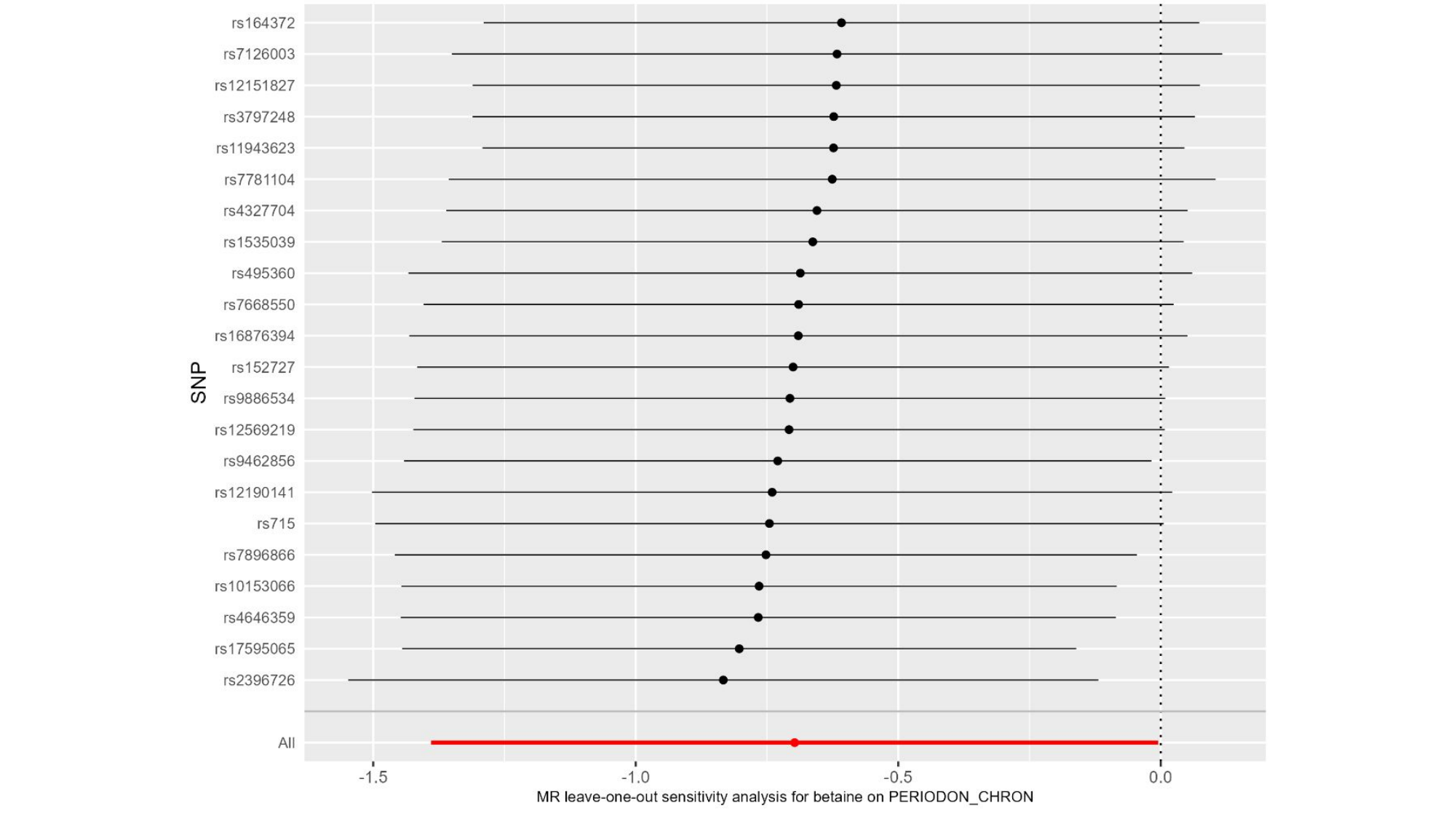

## Slide 7
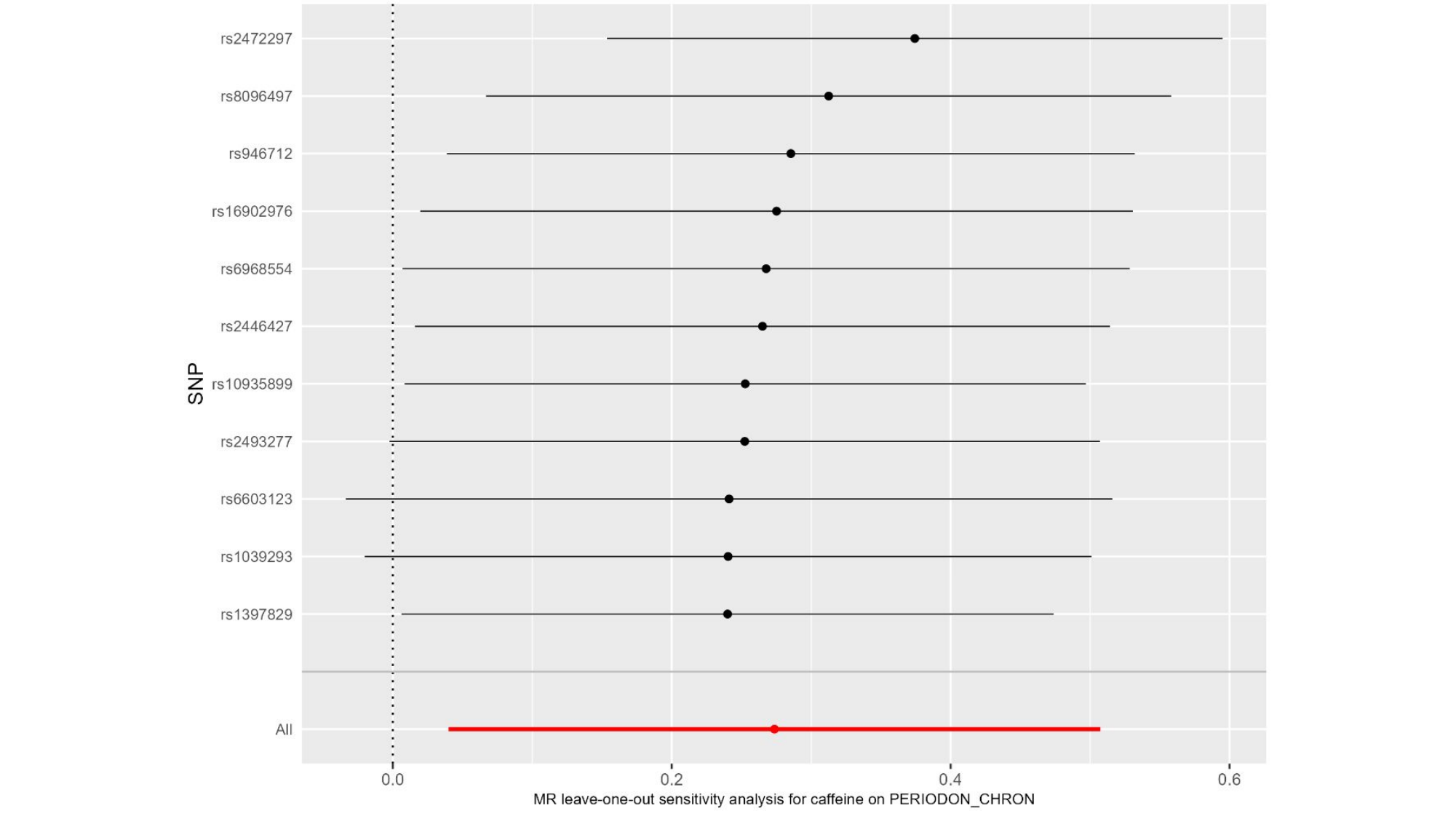

## Slide 8
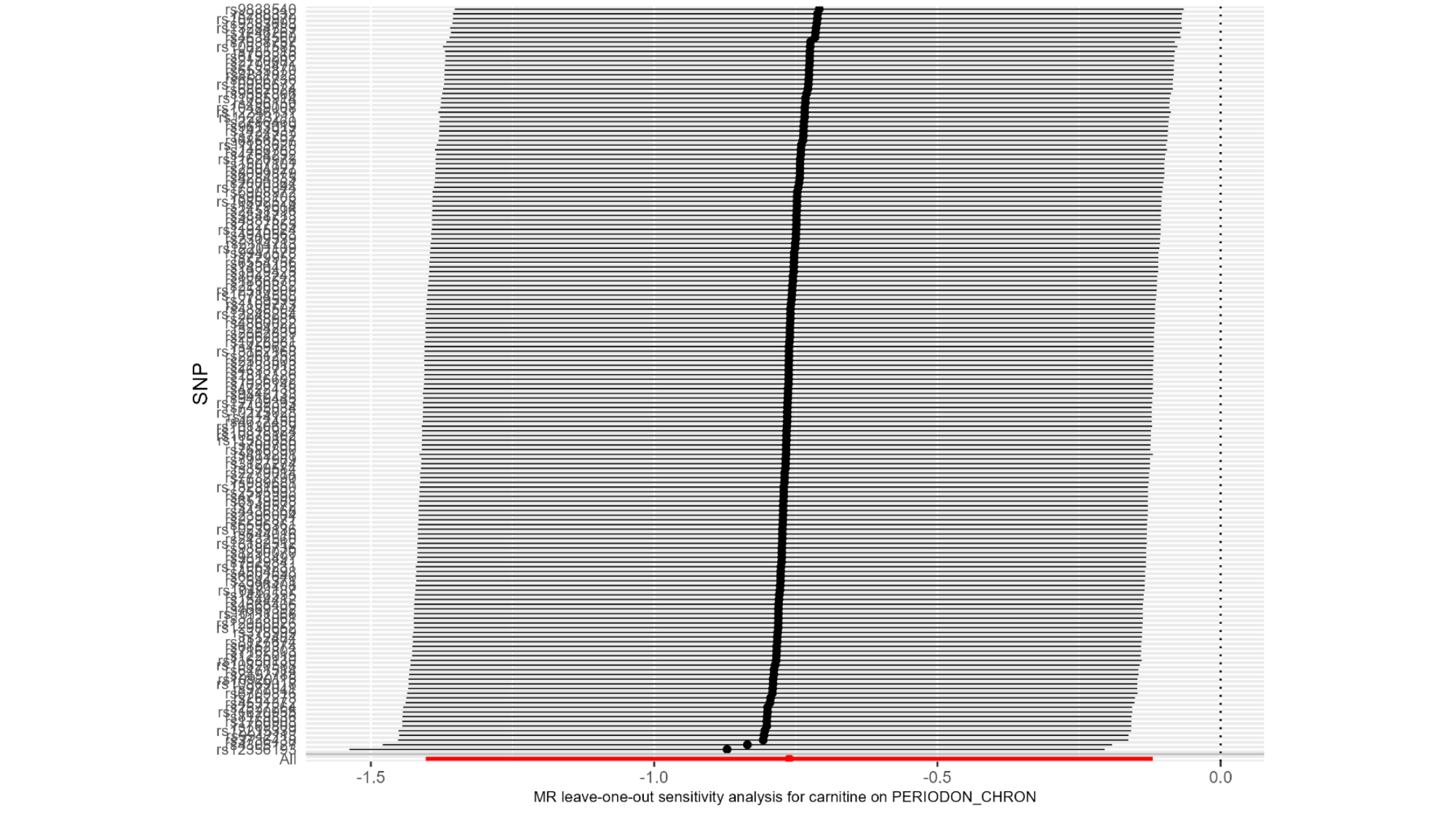

## Slide 9
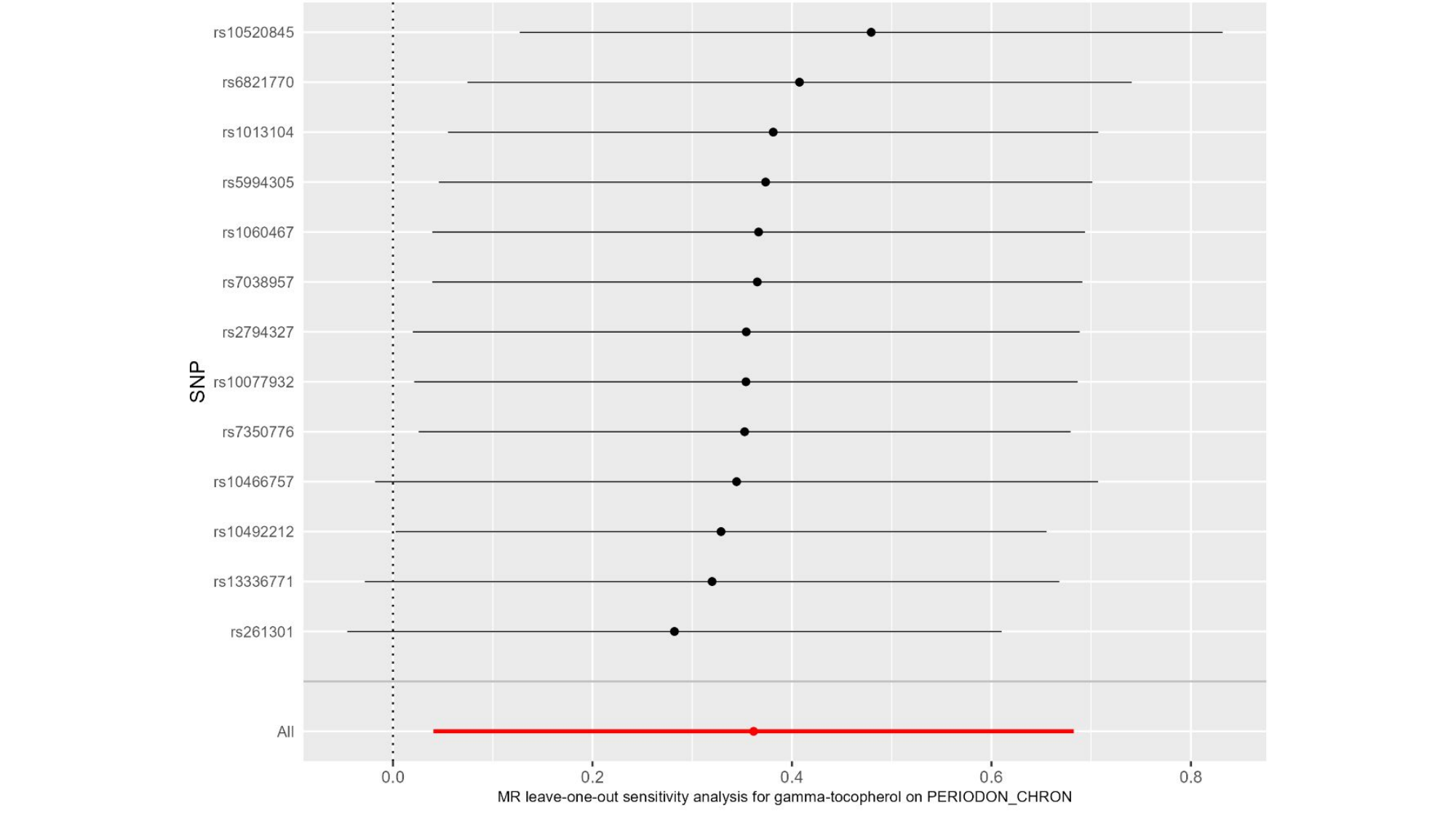

## Slide 10
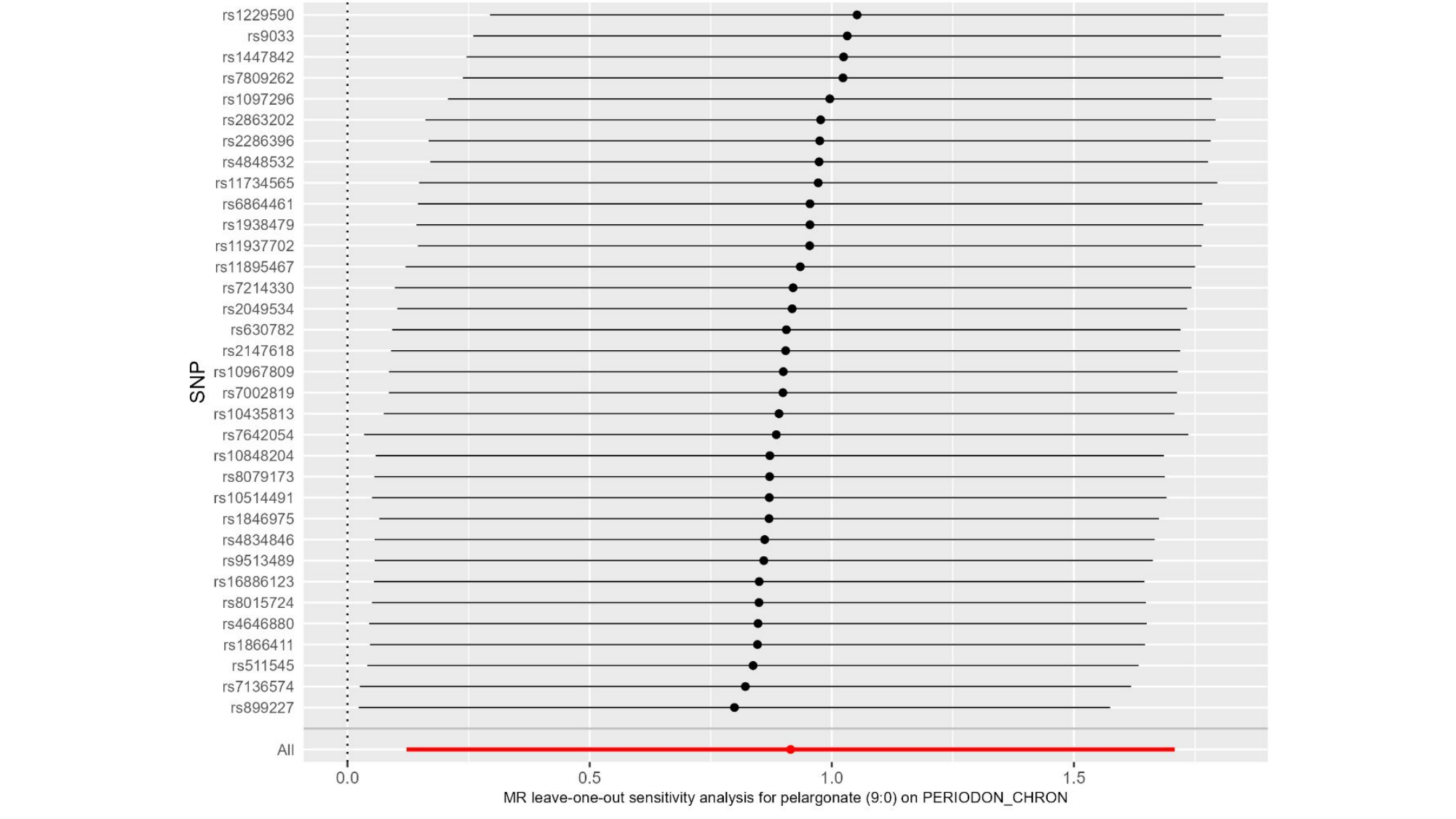

## Slide 11
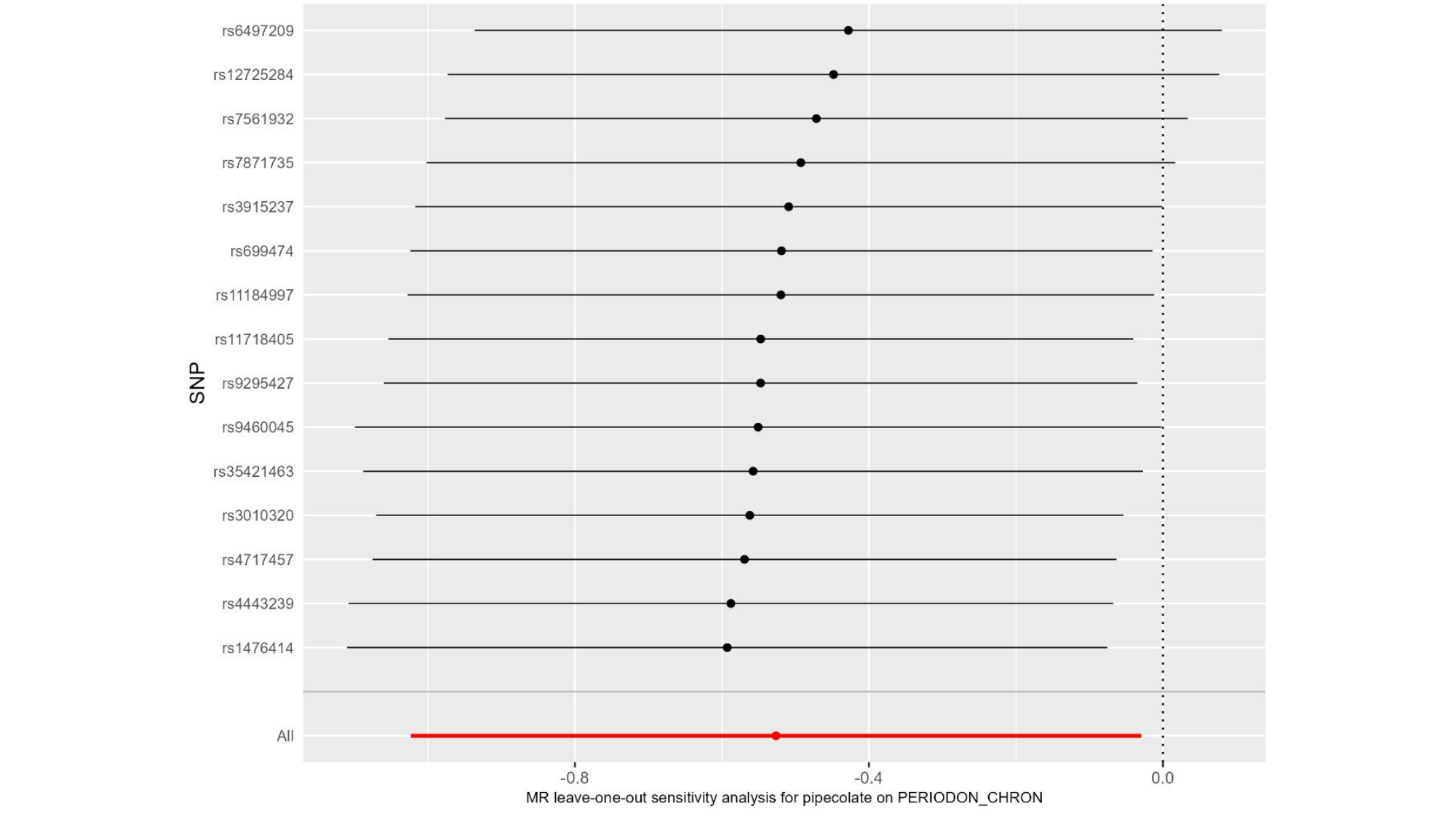

## Slide 12
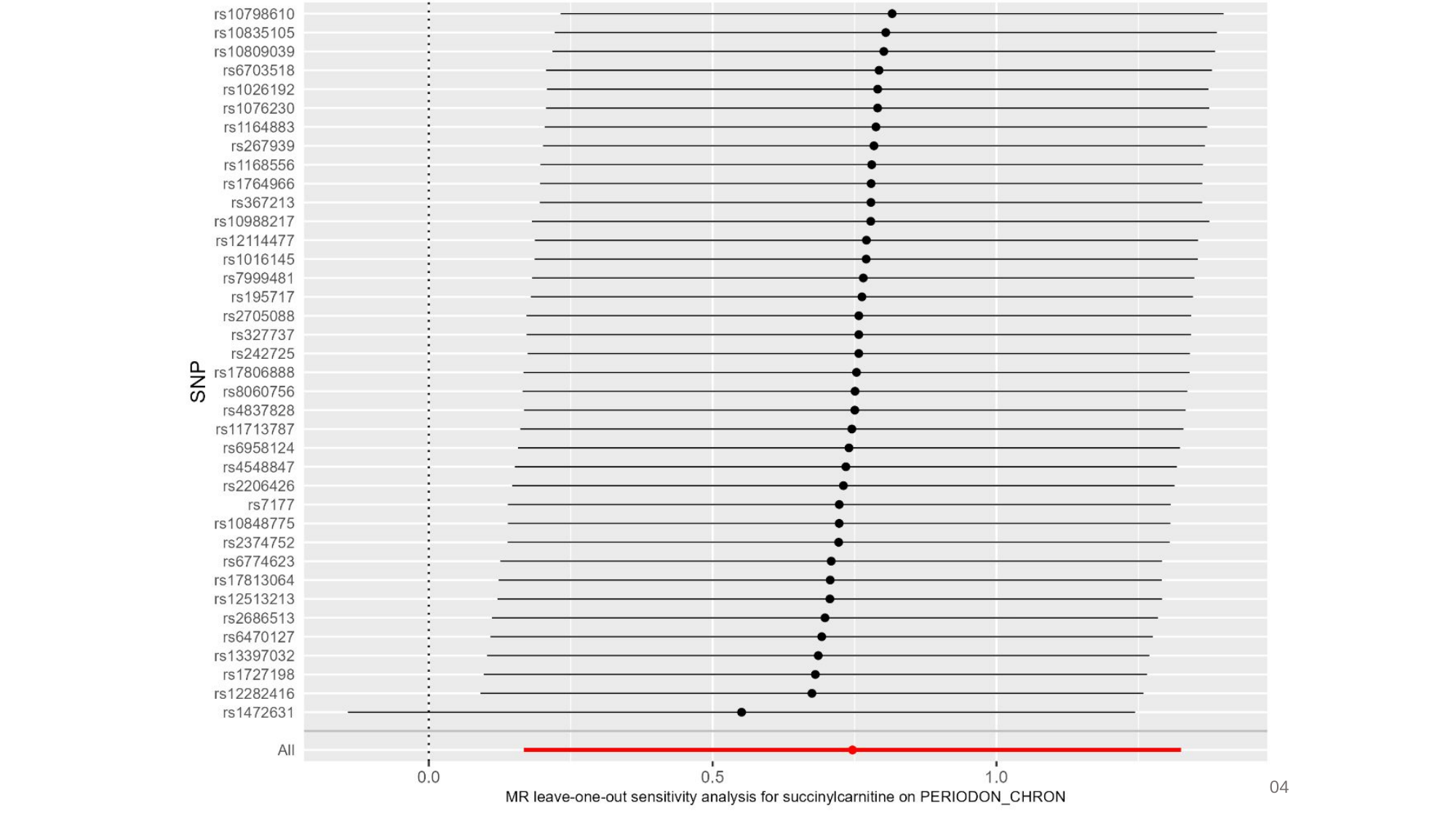

0.04

## Slide 13
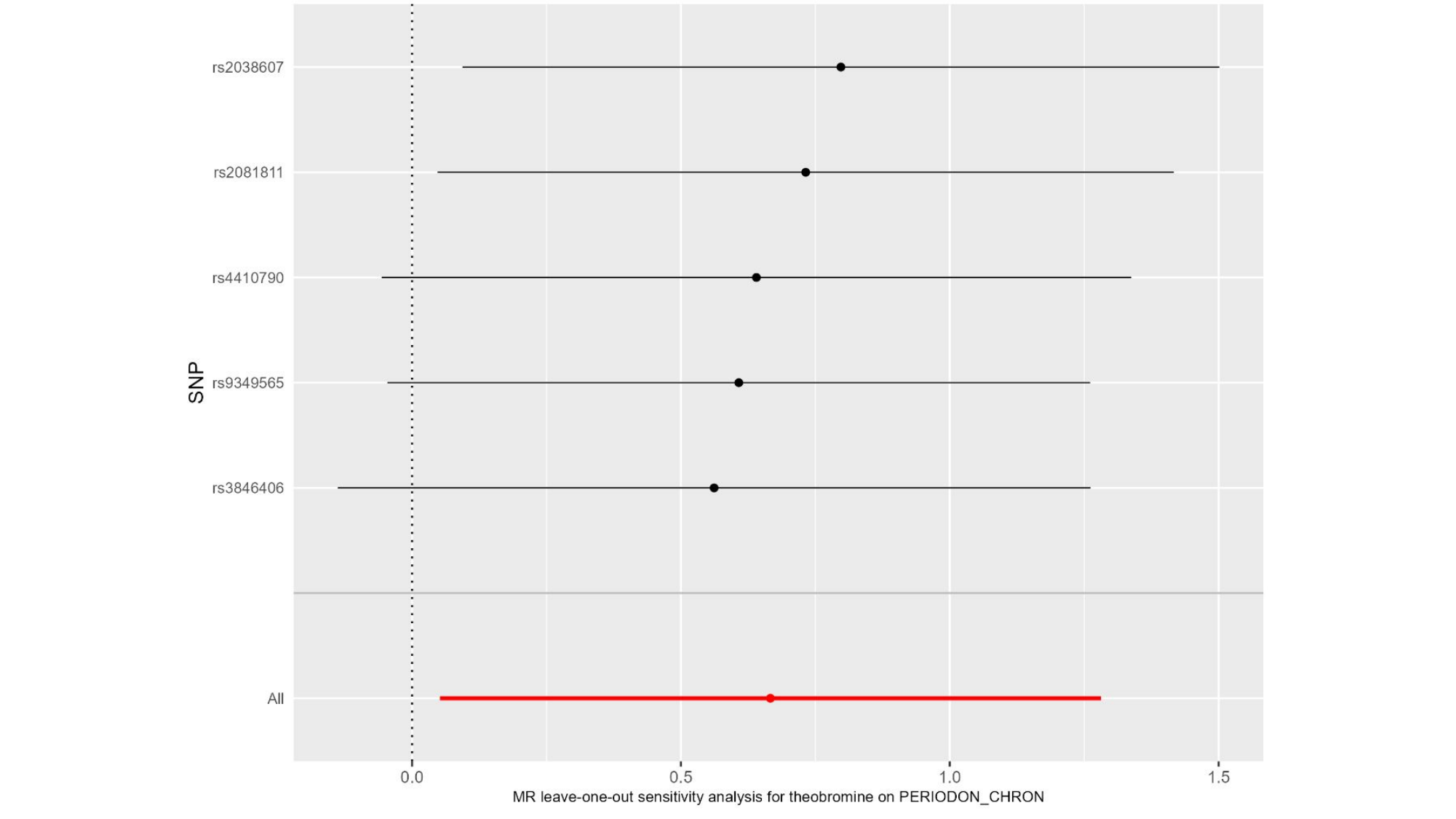

## Slide 14
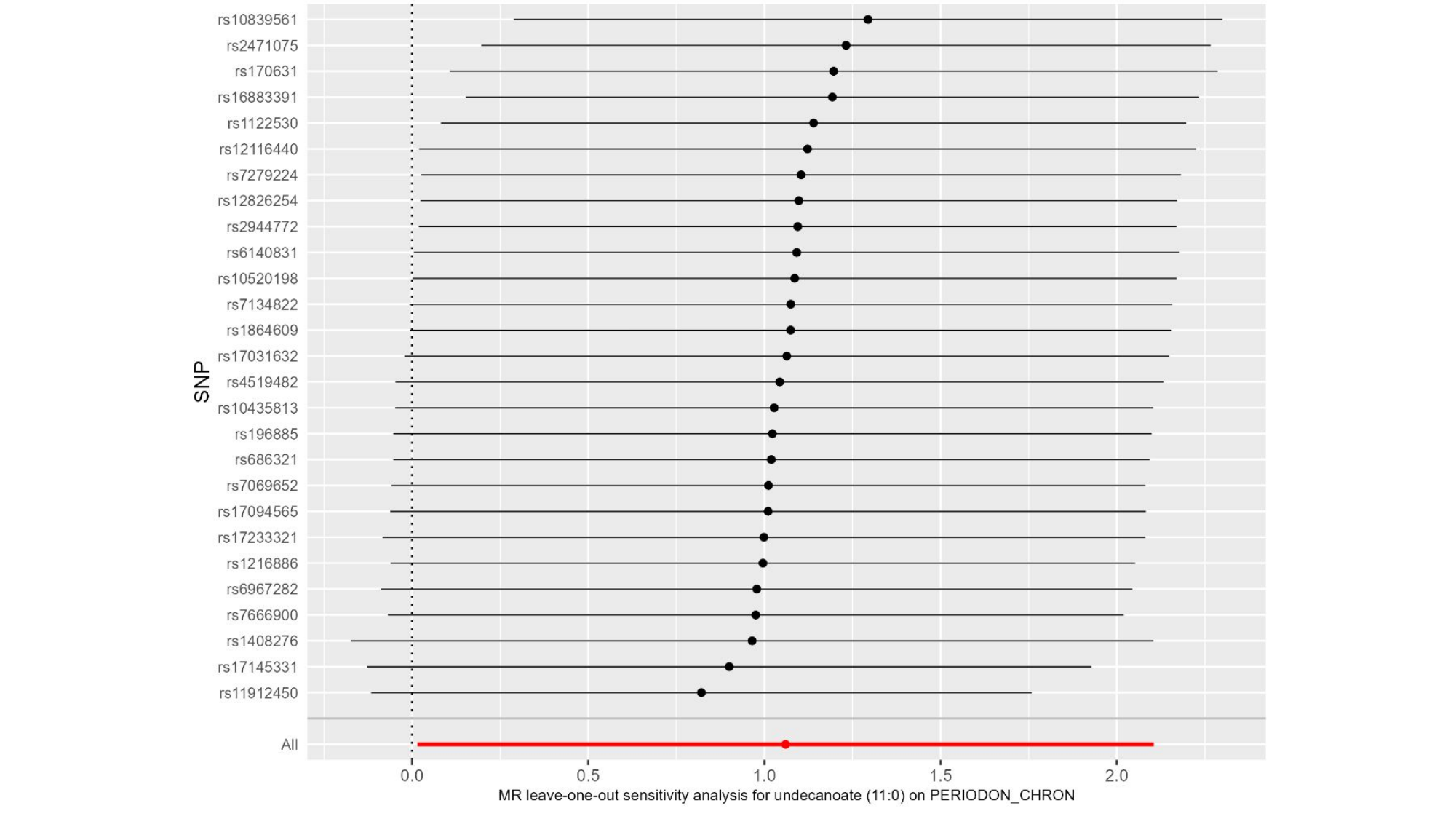

## Slide 15
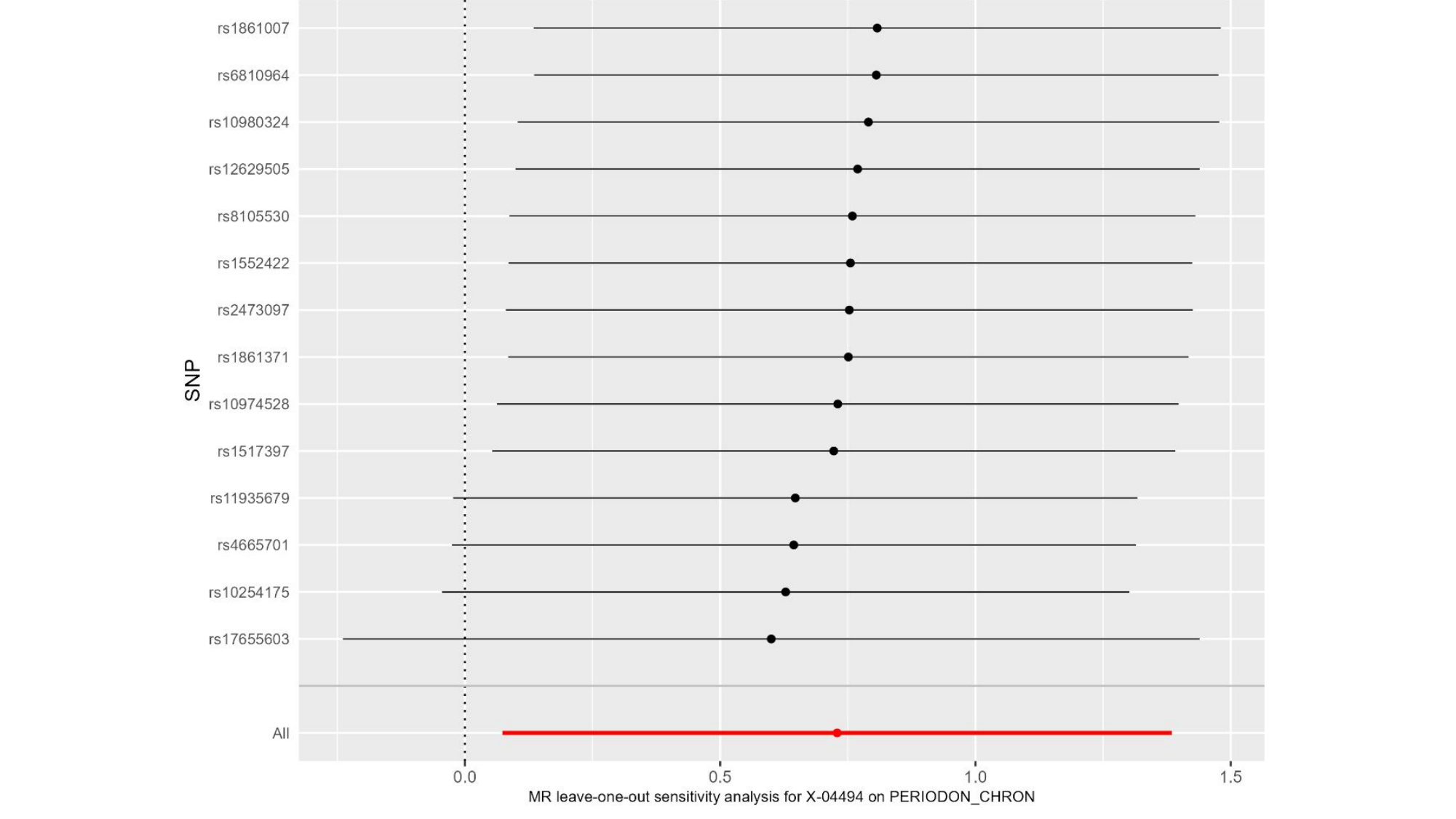

## Slide 16
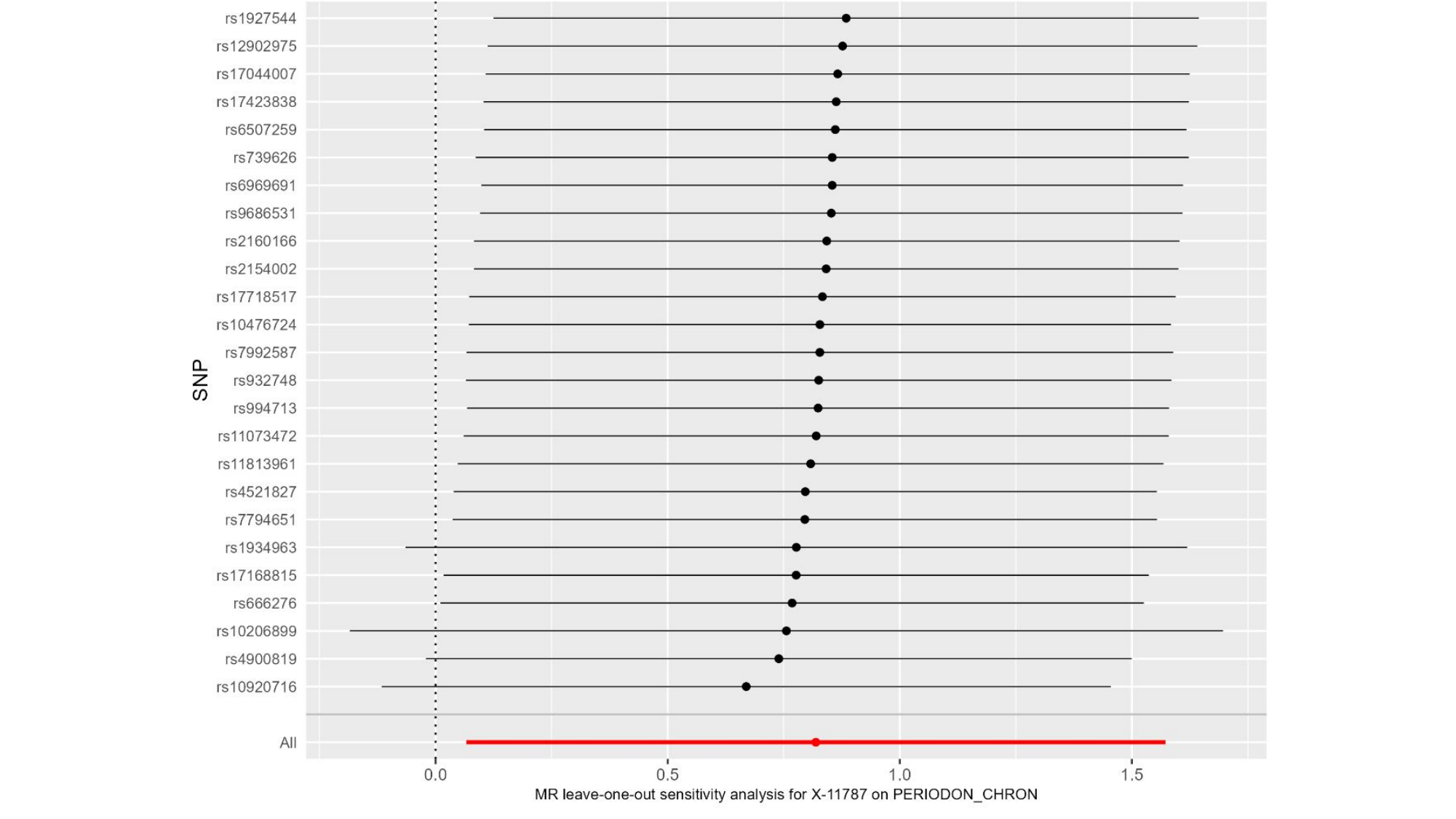

## Slide 17
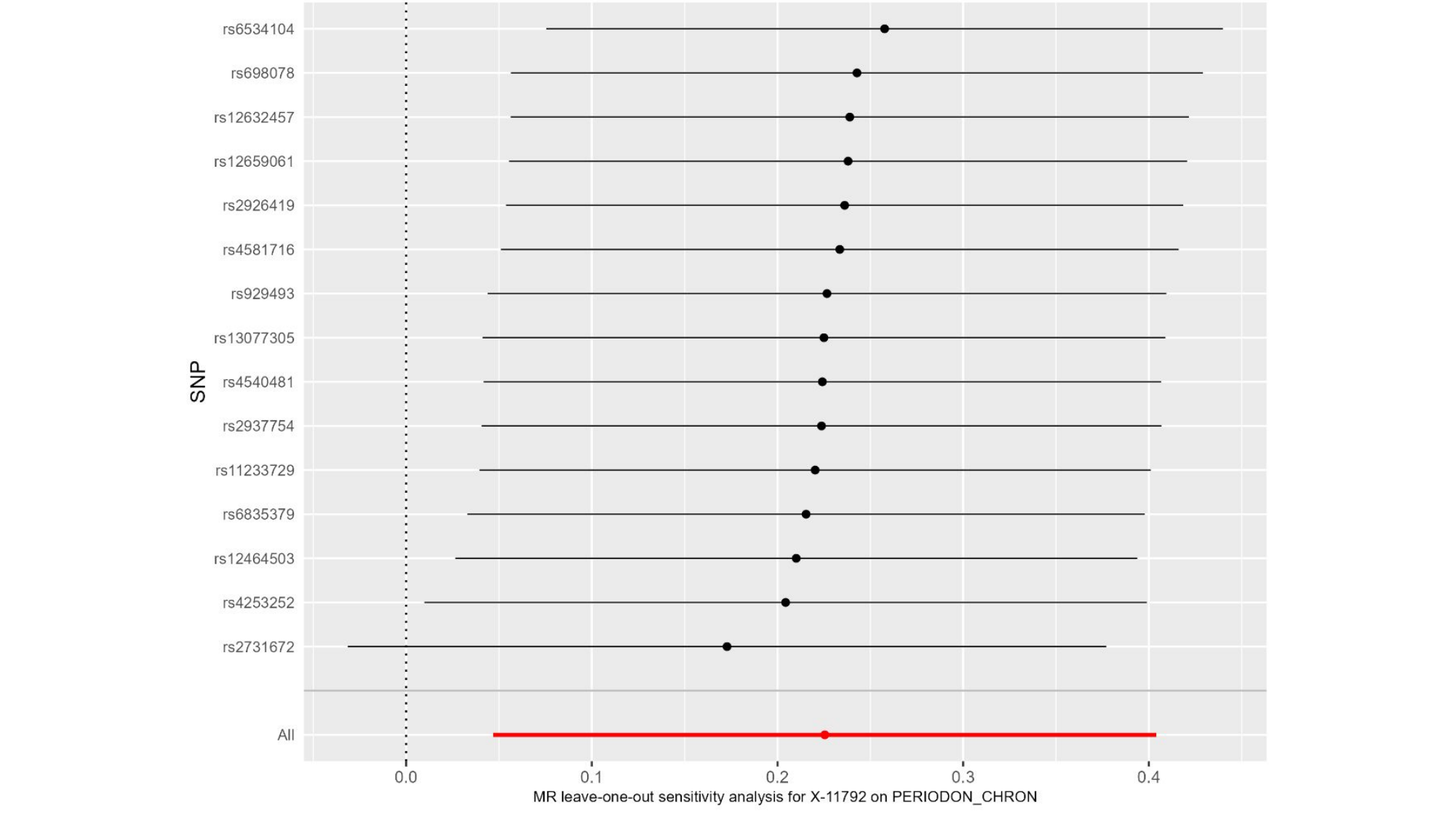

## Slide 18
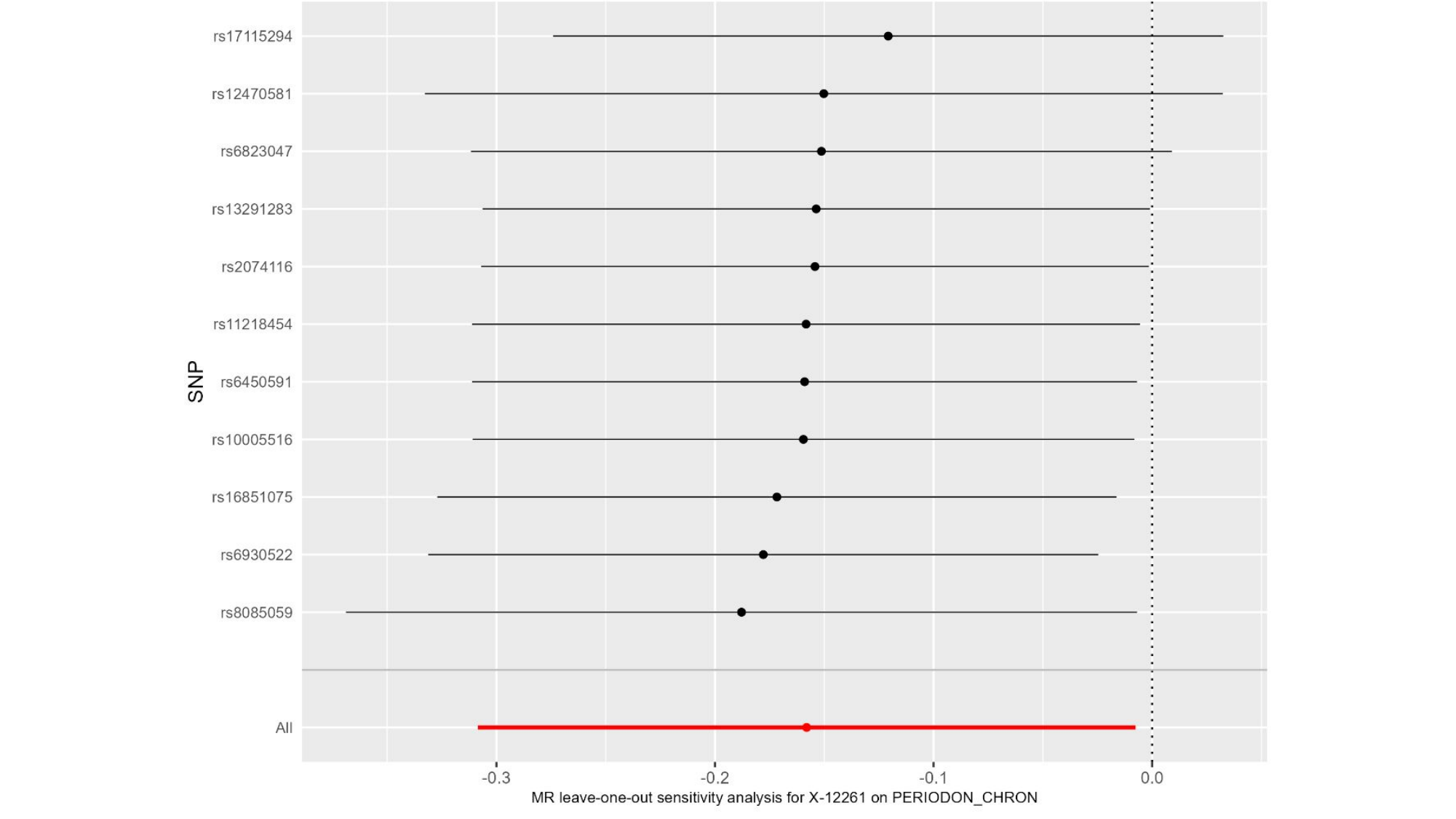

## Slide 19
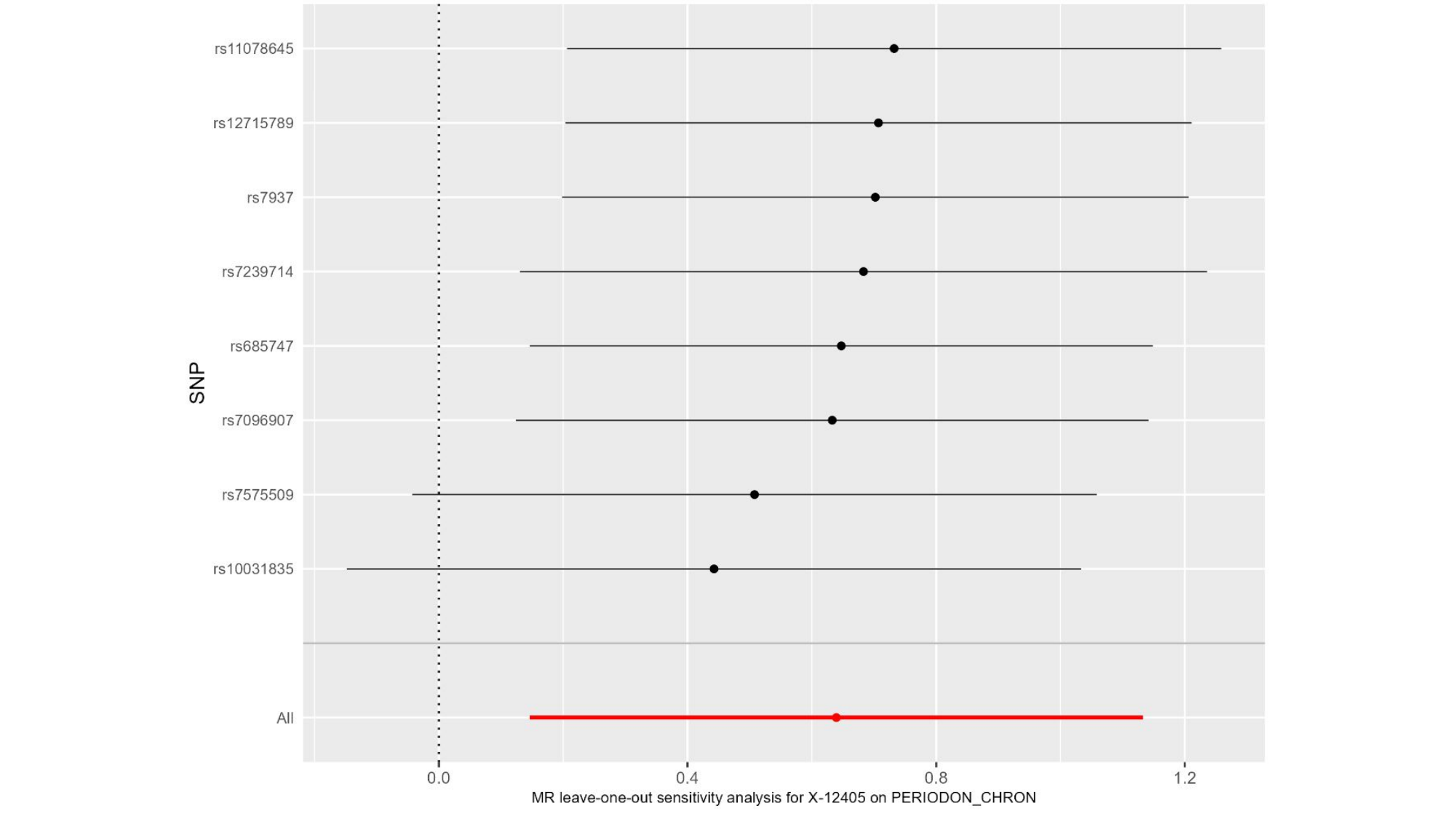

## Slide 20
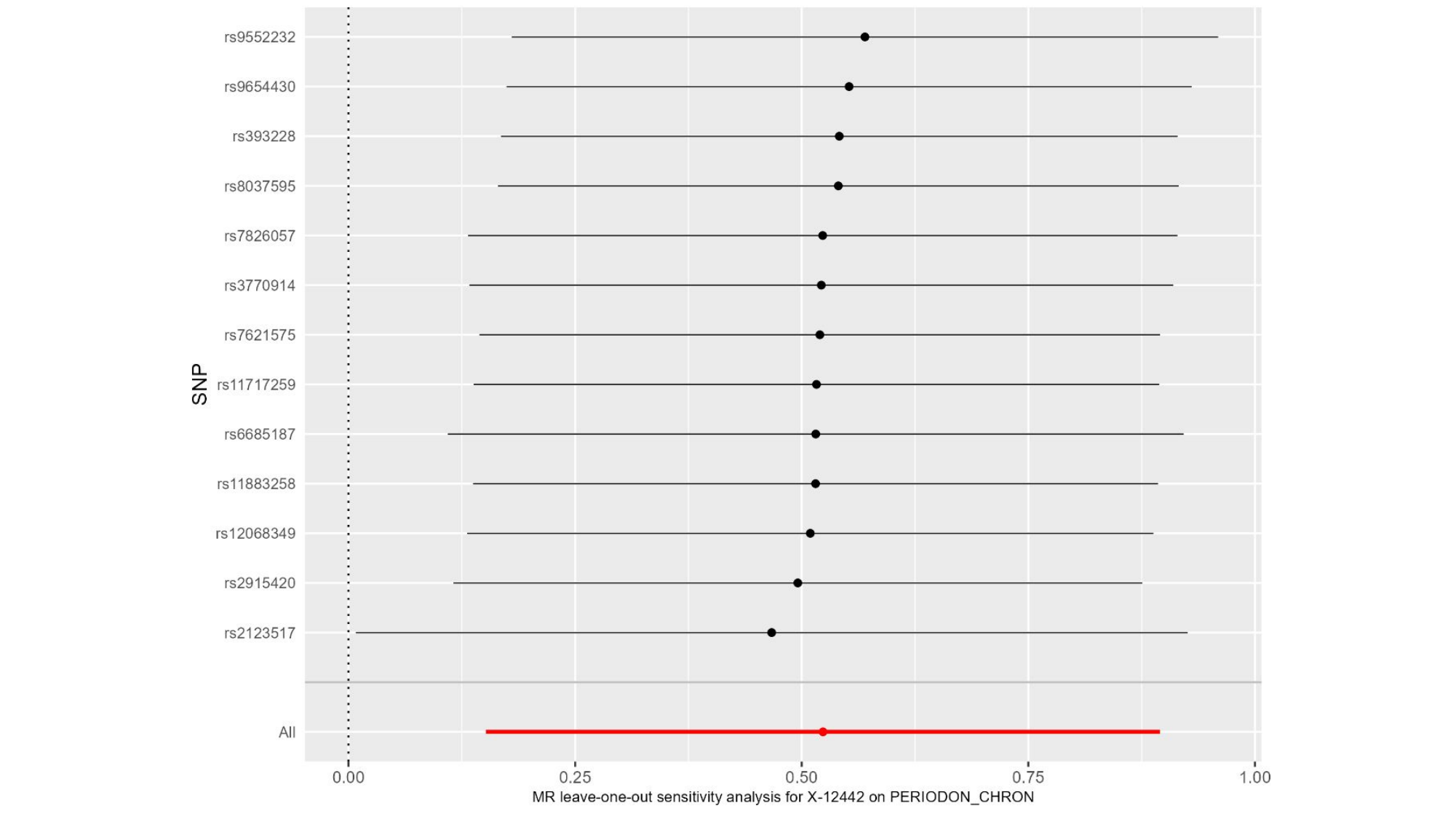

Supplement: Supplementary file 3 [file medi-105-e48615-s003.pptx]
