# Supplementary material for: Causal role of serum metabolites in chronic periodontitis: A bidirectional Mendelian randomization and multi-omics integration study
Source: Medicine (Baltimore). 2026 May 8;105(19):e48615. doi: 10.1097/MD.0000000000048615 (PMC13166884; doi:10.1097/MD.0000000000048615)
Supplement: Supplementary file 4 [file medi-105-e48615-s004.docx]

Supplementary Table S1. The reverse MR analysis between CP and 22 serum metabolites.

| Metabolite | nSNP |  | IVW | | MR Egger | | Simple mode | | Weighted median | | Weighted mode | |
| --- | --- | --- | --- | --- | --- | --- | --- | --- | --- | --- | --- | --- |
|  |  |  | b | pval | b | pval | b | pval | b | pval | b | pval |
| 1,5-anhydroglucitol (1,5-AG) | 27 |  | 0.0007 | 0.9470 | 0.0029 | 0.9247 | -0.0106 | 0.5851 | -0.0065 | 0.6176 | -0.0085 | 0.6138 |
| 1-stearoylglycerophosphoethanolamine | 12 |  | -0.0120 | 0.3288 | -0.0182 | 0.6354 | -0.0046 | 0.8350 | -0.0110 | 0.4714 | -0.0036 | 0.8677 |
| 3-indoxyl sulfate | 5 |  | -0.0293 | 0.0233 | -0.0354 | 0.3897 | -0.0323 | 0.2277 | -0.0334 | 0.0559 | -0.0363 | 0.1347 |
| 3-methylhistidine | 8 |  | -0.0212 | 0.4685 | -0.1523 | 0.1338 | -0.0132 | 0.8358 | -0.0212 | 0.5728 | -0.0742 | 0.2273 |
| 4-acetamidobutanoate | 33 |  | 0.0023 | 0.7457 | -0.0195 | 0.3903 | 0.0013 | 0.9025 | -0.0006 | 0.9444 | 0.0002 | 0.9844 |
| betaine | 22 |  | 0.0056 | 0.4605 | 0.0135 | 0.5706 | 0.0069 | 0.6123 | 0.0068 | 0.4624 | 0.0073 | 0.5680 |
| caffeine | 11 |  | -0.0149 | 0.6178 | -0.0224 | 0.8085 | 0.0136 | 0.8038 | -0.0016 | 0.9654 | 0.0050 | 0.9194 |
| carnitine | 159 |  | 0.0026 | 0.5280 | 0.0029 | 0.8296 | 0.0050 | 0.5320 | 0.0030 | 0.5658 | 0.0036 | 0.5882 |
| gamma-tocopherol | 13 |  | 0.0301 | 0.1808 | 0.0591 | 0.4407 | 0.0241 | 0.5109 | 0.0341 | 0.1406 | 0.0329 | 0.2584 |
| glycerol 3-phosphate (G3P) | 13 |  | -0.0164 | 0.0470 | -0.0090 | 0.7227 | -0.0196 | 0.2239 | -0.0176 | 0.0911 | -0.0180 | 0.2125 |
| pelargonate (9:0) | 34 |  | -0.0099 | 0.2749 | -0.0107 | 0.7264 | -0.0258 | 0.1989 | -0.0208 | 0.0347 | -0.0253 | 0.0922 |
| phenylalanine | 5 |  | -0.0022 | 0.6448 | 0.0139 | 0.3636 | -0.0068 | 0.4797 | -0.0028 | 0.6500 | -0.0041 | 0.6340 |
| pipecolate | 15 |  | 0.0166 | 0.2000 | -0.0029 | 0.9420 | 0.0245 | 0.3443 | 0.0195 | 0.2112 | 0.0233 | 0.3192 |
| succinylcarnitine | 38 |  | -0.0036 | 0.6862 | -0.0167 | 0.5818 | 0.0147 | 0.4564 | -0.0127 | 0.1971 | -0.0177 | 0.2187 |
| theobromine | 5 |  | -0.0094 | 0.6706 | -0.0509 | 0.4945 | -0.0100 | 0.8167 | -0.0084 | 0.7759 | -0.0133 | 0.7754 |
| undecanoate (11:0) | 27 |  | 0.0017 | 0.7305 | 0.0006 | 0.9678 | 0.0018 | 0.8475 | 0.0022 | 0.7222 | 0.0038 | 0.6600 |
| X-04494 | 14 |  | -0.0055 | 0.6100 | -0.0448 | 0.1869 | 0.0149 | 0.5078 | 0.0062 | 0.6664 | 0.0149 | 0.6059 |
| X-11787 | 25 |  | 0.0016 | 0.7710 | 0.0211 | 0.2531 | -0.0063 | 0.5873 | -0.0005 | 0.9334 | -0.0026 | 0.7794 |
| X-11792 | 15 |  | 0.0238 | 0.4661 | 0.1096 | 0.3082 | 0.0110 | 0.8682 | 0.0331 | 0.4455 | 0.0293 | 0.6209 |
| X-12261 | 11 |  | -0.0016 | 0.9624 | 0.0604 | 0.5820 | -0.0284 | 0.6496 | 0.0013 | 0.9771 | 0.0278 | 0.6640 |
| X-12405 | 8 |  | 0.0041 | 0.7600 | -0.0398 | 0.3545 | -0.0120 | 0.6383 | -0.0102 | 0.5637 | -0.0129 | 0.5499 |
| X-12442 | 13 |  | -0.0146 | 0.3040 | 0.0182 | 0.6827 | -0.0036 | 0.8906 | -0.0048 | 0.7976 | 0.0010 | 0.9649 |
