# Supplementary material for: Causal role of serum metabolites in chronic periodontitis: A bidirectional Mendelian randomization and multi-omics integration study
Source: Medicine (Baltimore). 2026 May 8;105(19):e48615. doi: 10.1097/MD.0000000000048615 (PMC13166884; doi:10.1097/MD.0000000000048615)
Supplement: Supplementary file 5 [file medi-105-e48615-s005.docx]

Supplementary Table S2. Metabolic pathway associated with CP.

| Pathway | Total | Expected | Hits | Raw p | FDR | Impact |
| --- | --- | --- | --- | --- | --- | --- |
| Caffeine metabolism | 10 | 0.0565 | 2 | 0.0012 | 0.1000 | 0.0000 |
| Lysine degradation | 30 | 0.1696 | 2 | 0.0114 | 0.4555 | 0.0000 |
| Phenylalanine, tyrosine and tryptophan biosynthesis | 4 | 0.0226 | 1 | 0.0224 | 0.5985 | 0.5000 |
| Phenylalanine metabolism | 8 | 0.0452 | 1 | 0.0444 | 0.8887 | 0.3571 |
| Histidine metabolism | 16 | 0.0905 | 1 | 0.0871 | 1.0000 | 0.0000 |
| Glycerolipid metabolism | 16 | 0.0905 | 1 | 0.0871 | 1.0000 | 0.0436 |
| One carbon pool by folate | 26 | 0.1470 | 1 | 0.1381 | 1.0000 | 0.0819 |
| Glycine, serine and threonine metabolism | 33 | 0.1866 | 1 | 0.1722 | 1.0000 | 0.0515 |
| Arginine and proline metabolism | 36 | 0.2035 | 1 | 0.1865 | 1.0000 | 0.0000 |
| Glycerophospholipid metabolism | 36 | 0.2035 | 1 | 0.1865 | 1.0000 | 0.0805 |
